# Prehospital transdermal glyceryl trinitrate in patients with ultra-acute presumed stroke (RIGHT-2): an ambulance-based, randomised, sham-controlled, blinded, phase 3 trial

**DOI:** 10.1016/S0140-6736(19)30194-1

**Published:** 2019-03-09

**Authors:** The RIGHT-2 Investigators, Philip M Bath, Polly Scutt, Craig S Anderson, Jason P Appleton, Evind Berge, Lesley Cala, Mark Dixon, Timothy M England, Peter J Godolphin, Diane Havard, Lee Haywood, Trish Hepburn, Kailash Krishnan, Grant Mair, Alan A Montgomery, Keith Muir, Stephen J Phillips, Stuart Pocock, John Potter, Chris Price, Marc Randall, Thompson G Robinson, Christine Roffe, Peter M Rothwell, Else C Sandset, Nerses Sanossian, Jeffrey L Saver, Angela Shone, A Niroshan Siriwardena, Joanna M Wardlaw, Lisa J Woodhouse, Graham Venables, Nikola Sprigg, Pierre Amarenco, Keith Muir, Shannon Amoils, Malcolm Jarvis, Peter M Rothwell, Peter Sandercock, Kjell Asplund, Colin Baigent, Sandeep Ankolekar, Harriet Howard, Christopher Lysons, Gemma Walker, Hayley Gregory, James Kirby, Jennifer Smithson, Joanne Keeling, Nadia Frowd, Robert Gray, Richard Dooley, Wim Clarke, Patricia Robinson, Zhe Kang Law, Sheila Hodgson, Adam Millington, Eleni Sakka, David Buchanan, Jeb Palmer, D Shaw, H Cobb, R Johnson, T Payne, R Spaight, A Spaight, M A Sajid, A Whileman, E Hall, H Cripps, J Toms, R Gascoyne, S Wright, M Cooper, A Palfreman, A Rajapakse, I Wynter, K Musarrat, A Mistri, C Patel, C Stephens, S Khan, S Patras, M Soliman, A Elmarimi, C Hewitt, E Watson, I Wahishi, J Hindle, L Perkin, M Wills, S Arif, S Leach, S Butler, D O'Kane, C Smith, J O'Callaghan, W Sunman, A Buck, B Jackson, C Richardson, G Wilkes, J Clarke, L Ryan, O Matias, D Mangion, A Hardwick, C Constantin, I Thomas, K Netherton, S Markova, A Hedstrom, B Rushton, C Hyde, J Scott, M Blair, M Maddula, R Donnelly, S Keane, S Johnson, H McKenzie, A Banerjee, D Hutchinson, H Goodhand, J Hill, K Mellows, M Cheeseman, V McTaggart, T Foster, L Prothero, P Saksena, A O'Kelly, H Wyllie, C Hacon, H Nutt, J North, K Goffin, J Potter, A Wiltshire, G Ravenhill, K Metcalf, L Ford, M Langley, W Davison, S Subramonian, F Magezi, I Obi, N Temple, N Butterworth-Cowin, P Oqwusu-Agyei, A F M Azim, A Nicolson, J Imam, J White, L Wood, R Fothergill, N Thompson, J Lazarus, H Werts, L Sztriha, C Ho, E McKenzie, E Owoyele, J Lim, J Aeron-Thomas, M Dockey, N Sylvester, P Rao, B M Bloom, E Erumere, G Norman, I Skene, L Cuenoud, L Howaniec, O Boulton, P Daboo, R Michael, S Al-Saadi, T Harrison, H Syed, L Argandona, S Amiani, R Perry, A Ashton, A Banaras, C Hogan, C Watchurst, E Elliott, N Francia, N Oji, R Erande, S Obarey, S Feerick, S Tshuma, E England, H Pocock, K Poole, S Manchanda, I Burn, S Dayal, K McNee, M Robinson, R Hancock, A South, C Holmes, A Steele, L B Guthrie, M Oborn, A Mohd Nor, B Hyams, C Eglinton, D Waugh, E Cann, N Wilmhurst, S Piesley, S Shave, D Dutta, M Obeid, D Ward, J Turfrey, J Glass, K Bowstead, L Hill, P Brown, S Beames, S O'Connell, V Hughes, R Whiting, J Gagg, M Hussain, M Harvey, D Karunatilake, B Pusuluri, A Witcher, C Pawley, J Allen, J Foot, J Rowe, C Lane, S Ragab, B Wadams, J Dube, B Jupp, A Ljubez, C Bagnall, G Hann, L Tucker, M Kelton, S Orr, F Harrington, A James, A Lydon, G Courtauld, K Bond, L Lucas, T Nisbett, J Kubie, A Bowring, G Jennings, K Thorpe, N Mason, S Keenan, L Gbadomishi, D Howcroft, H Newton, J Choulerton, J Avis, L Shaw, P Paterson, P Kaye, S Hierons, S Lucas, P Clatworthy, B Faulkner, L Rannigan, R Worner, B Bhaskaran, A Saulat, H Bearne, J Garfield-Smith, K Horan, P Fitzell, S Szabo, M Haley, D Simmons, D Cotterill, G Saunders, H Dymond, S Beech, K Rashed, A Tanate, C Buckley, D Wood, L Matthews, S Board, T Pitt-Kirby, N Rees, C Convery, P Jones, C Bryant, H Tench, M Dixon, R Loosley, S Coetzee, S Jones, T Sims, M Krishnan, C Davies, L Quinn, L Connor, M Wani, S Storton, S Treadwell, T Anjum, C Somashekar, A Chandler, C Triscott, L Bevan, M Sander, S Buckle, W Sayed, K Andrews, L Hughes, R Hughes, M Ward, A Pretty, A Rosser, B Davidson, G Price, I Gunson, J Lumley-Holmes, J Miller, M Larden, M Jhamat, P Horwood, R Boldy, C Jenkins, F Price, M Harrison, T Martin, N Ahmad, A Willberry, A Stevens, K Fotherby, A Barry, A Remegoso, F Alipio, H Maquire, J Hiden, K Finney, R Varquez, S Ispoglou, A Hayes, D Gull, R Evans, E Epstein, S Hurdowar, J Crossley, J Miles, K Hird, R Pilbery, C Patterson, H Ramadan, K Stewart, O Quinn, R Bellfield, S Macquire, W Gaba, A Nair, A Wilson, C Hawksworth, I Alam, J Greig, M Robinson, P Gomes, P Rana, Z Ahmed, P Anderston, A Neal, D Walstow, R Fong, S Brotheridge, A Bwalya, A Gillespie, C Midgley, C Hare, H Lyon, L Stephenson, M Broome, R Worton, S Jackson, R Rayessa, A Abdul-Hamid, C Naylor, E Clarkson, A Hassan, D Waugh, E Veraque, L Finch, L Makawa, M Carpenter, P Datta, A Needle, L Jackson, H J Brooke, J Ball, T Lowry, S Punnoose, R Walker, V Murray, A Ali, C Kamara, C Doyle, E Richards, J Howe, K Dakin, K Harkness, R Lindert, P Wanklyn, P Willcoxson, P Clark-Brown, R Mir

## Abstract

**Background:**

High blood pressure is common in acute stroke and is a predictor of poor outcome; however, large trials of lowering blood pressure have given variable results, and the management of high blood pressure in ultra-acute stroke remains unclear. We investigated whether transdermal glyceryl trinitrate (GTN; also known as nitroglycerin), a nitric oxide donor, might improve outcome when administered very early after stroke onset.

**Methods:**

We did a multicentre, paramedic-delivered, ambulance-based, prospective, randomised, sham-controlled, blinded-endpoint, phase 3 trial in adults with presumed stroke within 4 h of onset, face-arm-speech-time score of 2 or 3, and systolic blood pressure 120 mm Hg or higher. Participants were randomly assigned (1:1) to receive transdermal GTN (5 mg once daily for 4 days; the GTN group) or a similar sham dressing (the sham group) in UK-based ambulances by paramedics, with treatment continued in hospital. Paramedics were unmasked to treatment, whereas participants were masked. The primary outcome was the 7-level modified Rankin Scale (mRS; a measure of functional outcome) at 90 days, assessed by central telephone follow-up with masking to treatment. Analysis was hierarchical, first in participants with a confirmed stroke or transient ischaemic attack (cohort 1), and then in all participants who were randomly assigned (intention to treat, cohort 2) according to the statistical analysis plan. This trial is registered with ISRCTN, number ISRCTN26986053.

**Findings:**

Between Oct 22, 2015, and May 23, 2018, 516 paramedics from eight UK ambulance services recruited 1149 participants (n=568 in the GTN group, n=581 in the sham group). The median time to randomisation was 71 min (IQR 45–116). 597 (52%) patients had ischaemic stroke, 145 (13%) had intracerebral haemorrhage, 109 (9%) had transient ischaemic attack, and 297 (26%) had a non-stroke mimic at the final diagnosis of the index event. In the GTN group, participants' systolic blood pressure was lowered by 5·8 mm Hg compared with the sham group (p<0·0001), and diastolic blood pressure was lowered by 2·6 mm Hg (p=0·0026) at hospital admission. We found no difference in mRS between the groups in participants with a final diagnosis of stroke or transient ischaemic stroke (cohort 1): 3 (IQR 2–5; n=420) in the GTN group versus 3 (2–5; n=408) in the sham group, adjusted common odds ratio for poor outcome 1·25 (95% CI 0·97–1·60; p=0·083); we also found no difference in mRS between all patients (cohort 2: 3 [2–5]; n=544, in the GTN group *vs* 3 [2–5]; n=558, in the sham group; 1·04 [0·84–1·29]; p=0·69). We found no difference in secondary outcomes, death (treatment-related deaths: 36 in the GTN group *vs* 23 in the sham group [p=0·091]), or serious adverse events (188 in the GTN group *vs* 170 in the sham group [p=0·16]) between treatment groups.

**Interpretation:**

Prehospital treatment with transdermal GTN does not seem to improve functional outcome in patients with presumed stroke. It is feasible for UK paramedics to obtain consent and treat patients with stroke in the ultra-acute prehospital setting.

**Funding:**

British Heart Foundation.

## Introduction

High blood pressure is common in acute stroke and is a predictor of poor outcome; however, large trials investigating lowering blood pressure have given variable results, and the management of high blood pressure in acute stroke remains unclear,^1^ although lowering blood pressure in intracerebral haemorrhage is recommended in hospital.^2^ Nitric oxide (NO) donors are candidate treatments for acute stroke because of their cerebral and systemic vasodilatory action, which leads to a reduction in blood pressure. Preclinical stroke studies^3^, ^4^ found that NO donors improved regional cerebral blood flow and reduced stroke lesion size if administered rapidly. Further, vascular NO concentrations are low in acute stroke and are associated with a poor outcome,^5^, ^6^ raising the possibility that supplementing NO might be beneficial.

Five randomised trials^7^, ^8^, ^9^, ^10^, ^11^ of an NO donor, transdermal glyceryl trinitrate (GTN; also known as nitroglycerin), in acute stroke showed that GTN lowered peripheral and central blood pressure, 24 h blood pressure, pulse pressure, and augmentation index. Conversely, GTN had no effect on middle cerebral artery blood flow velocity, cerebral blood flow, intracranial pressure, or platelet function.^7^, ^8^, ^9^ Although four of the trials^7^, ^8^, ^9^, ^11^ were neutral for functional outcome, GTN improved functional outcome in the phase 2 Rapid Intervention with Glyceryl trinitrate in Hypertensive stroke Trial (RIGHT),^10^ with randomisation by paramedics within 4 h of stroke, and in a prespecified subgroup analysis of the phase 3 hospital-based Efficacy of Nitric Oxide in Stroke trial (ENOS),^11^, ^12^ with randomisation within 6 h of stroke. Summary and individual patient data meta-analyses^13^, ^14^ of these five trials suggested that very early administration of GTN within 6 h of onset (n=312) was beneficial in both ischaemic stroke and intracerebral haemorrhage, and reduced death, disability, cognitive impairment, mood disturbance, and poor quality of life. Beyond 6 h, treatment effects were neutral.

**Research in context**

**Evidence before this study**

We searched PubMed, Embase, and Web of Science for relevant articles on Sept 12, 2018, using the search terms “stroke”, “cerebrovascular accident”, “nitric oxide donor”, and “randomised controlled trial”. We also manually searched original articles and reviews in our own reference library. Searches were restricted to completed trials in humans with abstracts or full texts published and relating to administration of glyceryl trinitrate (GTN) within 6 h of stroke onset, and in which information on functional outcome and death was available. When combining results from two randomised controlled patient-masked trials with blinded-outcome assessment, one a pilot ambulance-based study and the other a prespecified subgroup of a large hospital-based trial, treatment with GTN within the first 6 h of stroke onset was associated with less death and reduced death or dependency, both overall and separately in ischaemic stroke and intracerebral haemorrhage.

**Added value of this study**

Ultra-acute administration of GTN in the ambulance within 4 h of stroke onset did not alter functional outcome in patients suspected to have stroke. It was feasible for UK paramedics to recruit, obtain consent, and treat patients with stroke in the prehospital environment.

**Implications of all the available evidence**

We did not find evidence that ultra-early administration of transdermal GTN improves functional outcome or reduces death in patients with suspected ultra-acute stroke. Large paramedic-delivered trials are possible in the UK.

For stroke interventions that do not require previous neuroimaging and that might have benefit in both ischaemic stroke and intracerebral haemorrhage, treatment before hospital admission will reduce time to initiation of treatment. The Field Administration of Stroke Therapy-Magnesium (FAST-MAG) ambulance-based stroke trial^15^ successfully recruited 1700 patients in the USA, but no previous large prehospital stroke trials have been completed in the UK.

We did the phase 3 RIGHT-2 trial to assess the safety and efficacy of GTN when given very early after presumed stroke onset by paramedics before participants were admitted to hospital. We also assessed the feasibility of performing a large multicentre, ambulance-based, paramedic-delivered trial in patients with presumed stroke in the UK.

## Methods

### Study design and participants

RIGHT-2 was a pragmatic, multicentre, paramedic-delivered, ambulance-based, prospective, randomised, sham-controlled, participant-blinded and outcome-blinded, phase 3 trial in adult participants with ultra-acute presumed stroke within 4 h of onset in the UK.

Adult patients were eligible for inclusion after an emergency telephone call (to 999 UK ambulance services) for presumed stroke if they presented within 4 h of onset of their symptoms to a trial-trained paramedic from a participating ambulance service and could be taken to a participating hospital. Patients had to have a face-arm-speech-time (FAST) score of 2 or 3 (thus ensuring the presence of motor weakness), and a systolic blood pressure of 120 mm Hg or higher. Patients from a nursing home, with reduced consciousness (Glasgow Coma Scale [GCS] score, <8 of 15), with hypoglycaemia (capillary glucose concentration <2·5 mmol/L), or who had a witnessed seizure were excluded (see appendix for a complete list of the inclusion and exclusion criteria).

Paramedics managed the primary consent process, and patients with capacity gave written informed consent that covered the whole trial. If capacity was absent, proxy consent was obtained from an accompanying relative, carer, or friend, if present, or from the paramedic if no accompanying person was present (as done in RIGHT).^10^ Confirmatory consent was obtained from the patient, or their relative, carer, or friend (if available) in hospital when the patient lacked capacity in the ambulance.

The final diagnosis was made after arrival to a participating hospital by the principal investigator based on clinical and neuroimaging findings and was categorised as intracerebral haemorrhage, ischaemic stroke, transient ischaemic attack, or non-stroke or transient ischaemic attack mimic.

The study was approved by the UK regulator (Medicines and Healthcare products Regulatory Agency, reference: 03057/0064/001–0001; Eudract 2015–000115–40) and national research ethics committee (IRAS: 167115) and was adopted by the National Institute for Health Research Clinical Research Network.

Details of the trial design, statistical analysis plan, and baseline data have been published,^16^, ^17^, ^18^ and the design and protocol are summarised in the appendix. The study protocol is available online.

### Randomisation and masking

Patients were enrolled and randomly assigned (1:1) by paramedics to receive transdermal GTN (5 mg as Transiderm-Nitro 5, Novartis, Frimley, UK; the GTN group) or a similar-appearing sham skin dressing (DuoDERM hydrocolloid dressing, Convatec, Flintshire, UK; the sham group). Randomisation was stratified by ambulance station with blocks of four packs (two active, two control) in a random permuted order. Each treatment pack was sealed to maintain blinding of paramedics. Ambulances carried only one pack at a time—paramedics signed-out the treatment pack with the lowest randomisation number from their ambulance station at the start of their shift and returned it if unused at the end of their shift. Opened but unused packs were returned to the coordinating centre. GTN patches or sham dressings came in marked sealed sachets so paramedics and nurses doing medication rounds in hospital knew treatment assignment. However, participants were effectively masked since the patches and dressings themselves were unlabelled, and a gauze dressing was taped over the top of the patch or dressing to provide additional masking.

### Procedures

The first treatment (GTN or sham) was administered by the paramedic immediately after randomisation in the ambulance, and further treatments were given to the patient for up to 3 days while in hospital. Patches or dressings were placed on the shoulder or back and the site changed daily.

Ambulance data (before and after first treatment) and hospital-collected clinical and neuroimaging data at admission (after first treatment), day 4 (end of treatment), and on death or discharge were entered online into a secure web-based database system. These data were then validated and used to confirm the patient's eligibility.

### Outcomes

The primary outcome was functional outcome assessed with the 7-level modified Rankin Scale (mRS), measured at 90 days after randomisation.^19^ mRS scores range from 0 to 6, with a score of 0 indicating no symptoms, 1 indicating some symptoms, 2–5 indicating increasing levels of disability and dependency, and 6 indicating death. Outcomes were recorded centrally by telephone by a trained assessor masked to treatment allocation; to ensure reliable scoring, raters used a structured questionnaire.^20^ If the participant could not be contacted by telephone (after multiple attempts), a questionnaire covering the same outcome measures was sent by post. The primary analysis involved a comparison of the distribution of all 7 levels of the mRS (shift) between the treatment groups.^21^

Participants were seen at day 4 (or at discharge, if earlier) to assess adherence to treatment and neurological deterioration (increase in the National Institutes of Health Stroke Scale [NIHSS] by at least 4 points from hospital admission to day 4 or worsening conscious level in the NIHSS consciousness domain item 1a). At discharge from hospital, duration of stay and discharge destination (to institution or home) were recorded. Prespecified secondary outcomes at day 90 included activities of daily living (Barthel Index), cognition (modified telephone Mini-Mental State Examination [t-MMSE], Telephone Interview for Cognition Scale-modified [TICS-M], categorical verbal fluency [with the use of animal naming]), health-related quality of life (European Quality of Life-5 dimensions-3 level [EQ-5D-3L], from which a health status utility value [HSUV] was calculated, EQ-visual analogue scale [EQ-VAS]), and mood (abbreviated Zung depression score [ZDS]) were recorded—all of which were used in ENOS.^11^

Safety outcomes included all-cause and cause-specific case fatality, hypotension or hypertension occurring during the first 4 days (as reported by investigators), and serious adverse events (all up to day 5, and fatal from day 5). Serious adverse events were validated and categorised by expert adjudicators who were masked to treatment assignment.

Plain brain scans (CT or MRI) performed on arrival at hospital were collected for central adjudication by expert neuroradiologists with the use of assessments updated from ENOS.^11^ Depending on local practice, CT or MR angiography was also performed and adjudicated centrally (see appendix for more information). Imaging outcomes on admission to hospital included infarct extent (International Stroke Trial-3 score, Alberta Stroke Program Early CT score), presence of hyperdense artery, haemorrhagic transformation, and mass effect, including midline shift for participants with ischaemic stroke, haematoma location, size, volume, extension (to subarachnoid spaces or ventricles), and mass effect, including midline shift for intracerebral haemorrhage, and type and location of mimics. On the next day, a research CT or MRI scan was done to assess safety; the same factors were assessed as above for ischaemic stroke and intracerebral haemorrhage.^22^

An independent Data Monitoring Committee reviewed unblinded data every 6 months and did a formal interim analysis midway through the trial (see appendix for description of the stopping rules); this analysis was done after 714 patients had been recruited and followed up at 90 days and the Data Monitoring Committee recommended that the trial should continue.

### Statistical analysis

We required a total sample size of 850 participants (425 in each group) to detect a shift in mRS with a common odds ratio [OR] of 0·70,^17^ assuming an overall significance level of 5%, 90% power, distribution of mRS scores as shown in the appendix,^10^ 3% loss to follow-up, mimic and transient ischaemic attack rate of 20%, and reduction for baseline covariate adjustment of 20%.^23^ During the trial, we noted the non-stroke diagnosis rate exceeded 30%. Since this mimic rate would reduce the number of participants recruited with a stroke diagnosis, and therefore the statistical power in this group, we increased the overall sample size from 850 to 1050 to maintain the overall effect size and statistical power. Further, a decision was made by the Trial Steering Committee to do a hierarchical analysis, comprising a sequential analysis done in two progressively inclusive cohorts based on the final in-hospital diagnosis: participants with confirmed stroke or transient ischaemic attack (cohort 1, target disease population) and stroke, transient ischaemic attack, or non-stroke or transient ischaemic attack (mimic)—ie, all patients (cohort 2, intention-to-treat [ITT]). Further information is given in the appendix.

We assessed the primary outcome using ordinal logistic regression with adjustment for age, sex, premorbid mRS, FAST score, baseline systolic blood pressure, index event (intracerebral haemorrhage, ischaemic stroke, transient ischaemic attack, mimic), time to randomisation, and reperfusion treatment (thrombectomy, alteplase, none).^17^ We tested the assumption of proportional odds using the likelihood ratio test. We assessed heterogeneity of the treatment effect on the primary outcome in prespecified subgroups by adding an interaction term to an adjusted ordinal logistic regression model. An unadjusted and per-protocol (as defined in the appendix) analysis is shown for completeness. We analysed death using Kaplan-Meier and adjusted Cox regression models. We assessed other outcomes using adjusted binary logistic regression (neurological deterioration, headache, hypotension, hypertension, feeding status, disposition, death in hospital), Cox regression (death), ordinal logistic regression (mRS, disposition), multiple linear regression (NIHSS, length of stay in hospital, t-MMSE, TICS-M, animal naming, ZDS, EQ-5D-HSUV, and EQ-VAS) and analysis of covariance (blood pressure). We analysed a global outcome (comprising ordered categorical or continuous data for mRS, Barthel Index, ZDS, TICS-M, and EQ-5D-HSUV) using the Wei-Lachin test.^24^ Participants who did not receive their assigned treatment, who did not adhere to the protocol, or who had a stroke mimic were still followed up in full at day 90 and are included in the main analyses. We made no adjustments for multiplicity of testing since all secondary analyses were hypothesis-generating and designed to support the primary analysis. We did primary analyses as randomised (cohort 2) using observed outcome data only with SAS software (version 9.4). In sensitivity analyses, we performed a per-protocol analysis, and missing mRS data were imputed using multiple regression-based imputation.

### Role of the funding source

This work was supported by the British Heart Foundation [grant number CS/14/4/30972] and sponsored by the University of Nottingham. There was no commercial support for the trial, and GTN patches and sham dressings were sourced by the Pharmacy at Nottingham University Hospitals NHS Trust. The funder of the study had no role in study design, data collection, data analysis, data interpretation, or writing of the report. The corresponding author and two statisticians (PS, LJW) had full access to all the data in the study and the corresponding author had final responsibility for the decision to submit for publication.

## Results

**Figure 1:**
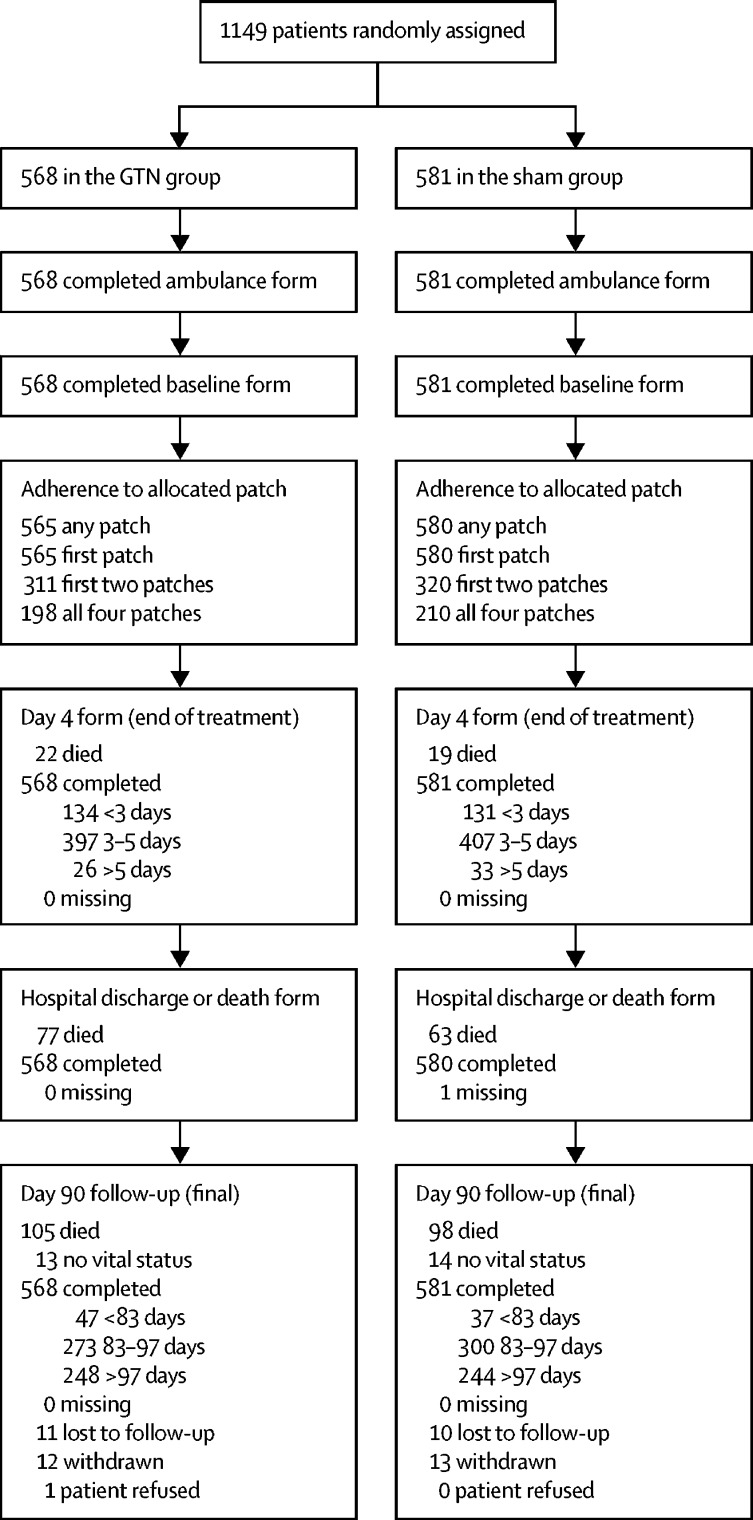
Trial profile for cohort 2 Cohort 2 includes all patients (intention-to-treat population). GTN=glyceryl trinitrate.

Between Oct 22, 2015, and May 23, 2018 (appendix), 1149 participants (cohort 2) were enrolled and randomly assigned (n=568 to the GTN group; n=581 to the sham group; figure 1) by 516 (35%) of 1492 trial-trained paramedics based at 184 ambulance stations in eight (62%) of 13 ambulance services in England and Wales; these participants were taken to 54 hospitals. For logistical reasons, screening logs were not kept. All patients gave consent in the ambulance, which was obtained from 603 (53%) patients, 429 (37%) relatives, carers, or friends, and 117 (10%) paramedics. Demographic and clinical characteristics were similar in the two treatment groups across cohort 1 and cohort 2 (table 1). The mean age was 72·5 years (SD 14·6), women comprised 48% of the participants, 60% of participants had a maximum FAST score of 3, and 26% had a GCS of less than 14. The final diagnosis of the qualifying event was 52% ischaemic stroke, 13% intracerebral haemorrhage, 9% transient ischaemic attack, and 26% stroke or transient ischaemic attack-mimicking condition. Common causes of stroke mimics included seizure (n=50 [18%]), migraine (n=49 [17%]), and functional symptoms (n=41 [14%]).

**Table 1:** Baseline patient characteristics in the ambulance and at hospital admission

		Patients with confirmed stroke or transient ischaemic attack (cohort 1)*		**All patients (cohort 2)**†	
		GTN group	Sham group	GTN group	Sham group
**Ambulance data (before randomisation)**					
Number of patients		434	418	568	581
Consent					
	Participant	220 (51%)	206 (49%)	296 (52%)	307 (53%)
	Relative, carer, or friend	169 (39%)	172 (41%)	213 (38%)	216 (37%)
	Paramedic	45 (10%)	40 (10%)	59 (10%)	58 (10%)
Age, years		73·7 (12·8)	75·3 (12·3)	72·3 (14·6)	72·7 (14·6)
Sex					
	Men	234 (54%)	220 (53%)	294 (52%)	300 (52%)
	Women	200 (46%)	198 (47%)	274 (48%)	281 (48%)
Time from onset to randomisation, min		70 (45–107)	70 (45–110)	70 (45–115)	72 (45–118)
Electrocardiogram, atrial fibrillation or flutter		81 (24%)	77 (22%)	92 (21%)	95 (20%)
Systolic blood pressure, mm Hg		163·4 (24·5)	163·0 (24·9)	161·5 (24·7)	162·8 (25·5)
Diastolic blood pressure, mm Hg		92·2 (19·1)	91·5 (17·8)	91·5 (18·5)	91·6 (17·2)
Heart rate, beats per min		81·6 (18·7)	82·2 (18·6)	81·7 (18·0)	82·6 (19·2)
Glasgow Coma Scale <14		123 (28%)	106 (25%)	162 (29%)	140 (24%)
FAST score of 3		276 (64%)	270 (65%)	343 (60%)	347 (60%)
**Hospital admission data (after randomisation)**					
Number of patients		434	418	568	581
Ethnic group, non-white		35 (8%)	43 (10%)	50 (9%)	63 (11%)
Premorbid mRS >2		76 (18%)	68 (16%)	115 (20%)	108 (19%)
Medical history					
	Hypertension	252 (58%)	249 (60%)	313 (56%)	330 (58%)
	Diabetes	82 (19%)	86 (21%)	109 (20%)	118 (21%)
	Previous stroke	100 (23%)	87 (21%)	137 (25%)	135 (24%)
	Ischaemic heart disease	66 (15%)	72 (17%)	95 (17%)	101 (18%)
	Current smoking	63 (18%)	51 (15%)	89 (19%)	79 (17%)
Qualifying event					
	Ischaemic stroke	302 (70%)	295 (71%)	302 (53%)	295 (51%)
	Intracerebral haemorrhage	74 (17%)	71 (17%)	74 (13%)	71 (12%)
	Stroke type unknown	1 (<1%)	0	1 (<1%)	0
	Transient ischaemic attack	57 (13%)	52 (12%)	57 (10%)	52 (9%)
	Non-stroke or transient ischaemic attack mimic	..	..	134 (24%)	163 (28%)

Data are n (%), mean (SD), and median (IQR). GTN=glyceryl trinitrate. FAST=face-arm-speech-time test. mRS=modified Rankin Scale.

* Target disease population.

† Intention-to-treat population.

The median time from the onset of symptoms to randomisation was 71 min (IQR 45–116) and to start of study drug 73 min (48–118) . Overall, the study drug was received within 30 min of symptom onset in 59 (5%) participants, within 60 min in 439 (38%) participants, and within 120 min in 865 (75%) participants.

Adherence to the first randomised treatment was excellent in both the confirmed stroke or transient ischaemic attack group (cohort 1: 849 (>99%) of target disease population) and in all participants (cohort 2: 1145 (>99%) of ITT population; appendix). In the per-protocol definition of adherence, which required that at least the first two doses of treatment were received, only 571 (67%) of cohort 1 and 631 (55%) of cohort 2 were adherent; common reasons for non-adherence were a diagnosis of non-stroke, early discharge, a medical decision to stop randomised treatment, a procedural error, or missing trial medication (appendix). Just 382 (45%) of participants with a stroke or transient ischaemic attack (cohort 1), and 408 (36%) of participants overall (cohort 2), received all 4 days of treatment.

There were 38 protocol violations in the ambulance and these mainly comprised inclusion of patients beyond 4 h, with a FAST score of less than 2, a systolic blood pressure of less than 120 mm Hg, or who were from a nursing home (appendix). The most common protocol violations in hospital involved not administering the second day's treatment or failure to obtain secondary consent.

In cohort 1, systolic blood pressure at baseline was 163·2 mm Hg (SD 24·7) and diastolic was 91·9 mm Hg (18·5; table 1) and reduced in both GTN and sham groups over the 4 days after randomisation (appendix). After treatment, systolic blood pressure reduced by 5·8 mm Hg (p<0·0001) at hospital admission and diastolic by 2·6 mm Hg (p=0·0026) in the GTN group compared with the sham group. At day 2, systolic blood pressure reduced by 5·3 mm Hg (p=0·00016) and diastolic by 2·6 mm Hg (p=0·0054) in the GTN group compared with the sham group. The difference in blood pressure between the GTN and sham groups then diminished with no difference at days 3 and 4. Similar findings were seen for the effect of GTN on blood pressure in all patients (cohort 2; appendix). In all patients, symptomatic hypotension was more common in the GTN group (21 [4%] patients) than in the sham group (9 [2%] patients; adjusted OR [aOR] 2·49 [95% CI 1·11–5·57]; p=0·026; table 2). Heart rate did not differ between the treatment groups (data not shown).

**Table 2:** Primary and secondary outcomes at day 4 and day 90 in cohort 1 and cohort 2

		Cohort 1*					**Cohort 2**†				
		Number of patients (n=852)	GTN group (n=434)	Sham group (n=418)	acOR, aOR, aDIM, or aHR (95% CI)	p value	Number of patients (n=1149)	GTN group (n=568)	Sham group (n=581)	acOR, aOR, aDIM, or aHR (95% CI)	p value
Day 90 mRS, maximum score of 6 (primary outcome)		828	3 (2–5)	3 (2–5)	1·25 (0·97 to 1·60)	0·083	1102	3 (2–5)	3 (2–5)	1·04 (0·84 to 1·29)	0·69
Sensitivity analyses											
	Unadjusted	828	3 (2–5)	3 (2–5)	1·05 (0·83 to 1·33)	0·70	1102	3 (2–5)	3 (2–5)	0·99 (0·81 to 1·22)	0·96
	Mean	828	3·4 (2·0)	3·4 (1·9)	0·14 (−0·07 to 0·36)	0·19	1102	3·2 (2·0)	3·2 (1·9)	0·01 (−0·17 to 0·19)	0·92
	mRS >2	828	286 (68%)	282 (69%)	1·11 (0·79 to 1·57)	0·55	1102	358 (66%)	373 (67%)	1·02 (0·76 to 1·38)	0·88
	Per protocol	714	3 (2–5)	3 (2–5)	1·22 (0·93 to 1·60)	0·14	959	3 (2–5)	3 (2–5)	1·05 (0·84 to 1·33)	0·65
	Imputed	852	3 (2–5)	3 (2–5)	1·23 (0·96 to 1·57)	0·10	1149	3 (2–5)	3 (2–5)	1·05 (0·85 to 1·30)	0·65
Hospital admission											
	NIHSS, maximum score of 42	755	10·5 (7·6)	10·4 (7·7)	0·34 (−0·51 to 1·19)	0·43	931	9·7 (7·6)	9·4 (7·5)	0·14 (−0·61 to 0·89)	0·72
	GCS, maximum score of 15	835	13·5 (2·3)	13·8 (2·0)	−0·37 (−0·64 to −0·10)	0·0068	1076	13·7 (2·2)	13·9 (1·9)	−0·19 (−0·42 to 0·04)	0·10
	FAST, maximum score of 3	799	2·3 (0·9)	2·2 (1·0)	0·09 (−0·02 to 0·19)	0·10	985	2·2 (1·0)	2·1 (1·0)	0·03 (−0·07 to 0·13)	0·51
	OCSP, TACS	822	161 (38%)	149 (37%)	1·13 (0·82 to 1·55)	0·45	1046	176 (34%)	174 (33%)	1·03 (0·78 to 1·37)	0·83
Day 4 (discharge)											
	Death	849	20 (5%)	18 (4%)	1·17 (0·57 to 2·39)	0·68	1128	22 (4%)	19 (3%)	1·19 (0·60 to 2·35)	0·63
	Neurological deterioration‡	534	60 (23%)	56 (21%)	1·14 (0·74 to 1·77)	0·56	586	62 (21%)	59 (20%)	1·08 (0·70 to 1·65)	0·73
	Headache§	843	41 (10%)	28 (7%)	1·41 (0·84 to 2·37)	0·19	1117	49 (9%)	36 (6%)	1·43 (0·90 to 2·27)	0·13
	Hypotension§	844	18 (4%)	9 (2%)	2·07 (0·90 to 4·75)	0·085	1118	21 (4%)	9 (2%)	2·49 (1·11 to 5·57)	0·026
	Hypertension§	844	89 (21%)	93 (22%)	0·82 (0·57 to 1·18)	0·28	1118	106 (19%)	108 (19%)	0·96 (0·69 to 1·33)	0·81
	Feeding: non-oral	806	123 (30%)	132 (33%)	0·89 (0·63 to 1·26)	0·51	1049	130 (25%)	139 (26%)	0·89 (0·65 to 1·24)	0·50
Events in hospital											
	Length of stay	847	17·4 (29·7)	19·1 (28·9)	−1·35 (−5·16 to 2·46)	0·49	1126	14·6 (27·0)	15·3 (25·7)	−1·09 (−4·00 to 1·81)	0·46
	Died	847	72 (17%)	60 (14%)	1·28 (0·84 to 1·96)	0·26	1126	78 (14%)	63 (11%)	1·35 (0·90 to 2·02)	0·15
	Died or in an institution	831	180 (42%)	167 (41%)	1·17 (0·84 to 1·61)	0·35	1102	193 (35%)	186 (33%)	1·08 (0·81 to 1·46)	0·60
Day 90											
	Death	841	97 (23%)	79 (19%)	1·24 (0·91 to 1·68)	0·17	1122	105 (19%)	98 (17%)	1·11 (0·84 to 1·47)	0·47
	Disposition, maximum score of 3¶	809	1 (1–2)	1 (1–2)	1·32 (0·96 to 1·82)	0·086	1069	1 (1–2)	1 (1–2)	1·11 (0·83 to 1·47)	0·48
	EQ-5D-HSUV, maximum score of 1‖	798	0·4 (0·4)	0·4 (0·4)	−0·02 (−0·07 to 0·03)	0·42	1055	0·4 (0·4)	0·4 (0·4)	0·00 (−0·04 to 0·05)	0·95
	Barthel Index, maximum score of 100^d^	795	56·2 (45·0)	57·5 (43·9)	−2·74 (−7·82 to 2·33)	0·29	1048	60·3 (43·7)	61·3 (43·1)	−0·24 (−4·54 to 4·06)	0·91
	TICS-M, maximum score of 39‖**	439	12·4 (12·3)	13·2 (12·1)	−0·87 (−2·63 to 0·90)	0·34	551	13·5 (12·3)	13·7 (11·8)	0·06 (−1·50 to 1·63)	0·94
	ZDS, maximum score of 100‖**	499	67·3 (29·7)	66·0 (29·1)	1·38 (−2·87 to 5·63)	0·52	638	66·5 (28·8)	65·1 (28·6)	0·53 (−3·22 to 4·28)	0·78
	Global outcome (MWD)	828	..	..	0·02 (−0·06 to 0·10)	0·62	1102	..	..	0·00 (−0·06 to 0·07)	0·92
	Home time, days‡	682	55·8 (49·2)	55·5 (46·8)	−0·30 (−6·14 to 5·54)	0·92	903	63·5 (48·9)	63·7 (46·9)	2·18 (−2·81 to 7·16)	0·39

Data are n (%), mean (SD), and median (IQR), unless otherwise stated. GTN=glyceryl trinitrate. acOR=adjusted common odds ratio. aOR=adjusted odds ratio. aDIM=adjusted difference in means. aHR=adjusted hazard ratio. mRS=modified Rankin scale. NIHSS=National Institutes of Health Stroke Scale. GSC=Glasgow Coma Scale. FAST=face-arm-speech-time test (calculated from NIHSS). OCSP=Oxford Community Stroke Project. TACS=total anterior circulation syndrome (in ischaemic stroke and intracerebral haemorrhage). EQ-5D-HSUV=Euro-Quality of life-5 Dimensions health status utility value. TICS-M=modified telephone interview cognition scale. ZDS=Zung depression scale. MWD=Mann-Whitney difference. EQ-VAS=Euro-Quality of life-Visual Analogue Scale. t-MMSE=telephone mini-mental state examination.

* Patients with confirmed stroke or transient ischaemic attack (modified intention-to-treat population).

† All patients (intention-to-treat population).

‡ Neurological deterioration from hospital admission: NIHSS ≥4 points or ≥2 point increase in any domain.

§ Clinical.

¶ Disposition: home (score of 1), institution or in hospital (score of 2), died (score of 3) by day 90.

‖ Death scored as: Barthel Index −5, verbal fluency (animal naming) −1, EQ-VAS −1, home time −1, t-MMSE −1, TICS-M −1, EQ-5D-HSUV 0, GCS 2, mRS 6, NIHSS 43, ZDS 102·5.

** Incomplete TICS-M and ZDS due to inability by participants with severe stroke to respond to questions.

Vital status was available in 1122 (98%) participants and mRS in 1102 (96%; figure 1); we found no differential loss to follow-up or withdrawals between the treatment groups. Masking was maintained with participants unable to identify which medication they had received (appendix).

**Figure 2:**
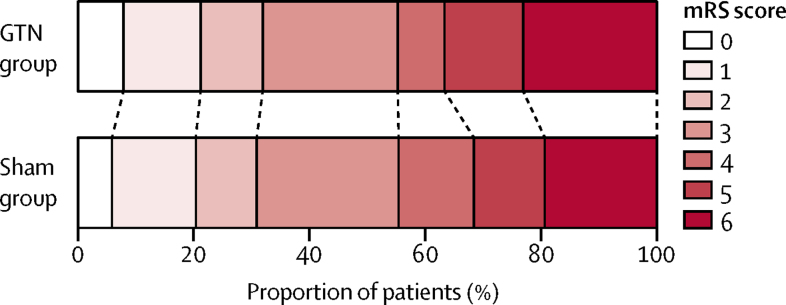
Distribution of mRS score at day 90 for GTN versus sham in cohort 1 Cohort 1 includes patients with confirmed stroke or transient ischaemic stroke (modified intention to treat). Comparison by ordinal logistic regression adjusted for age, sex, premorbid mRS, FAST score, pretreatment systolic blood pressure, index event (intracerebral haemorrhage, ischaemic stroke, transient ischaemic attack, mimic), and time to randomisation. GTN=glyceryl trinitrate. mRS=modified Rankin Scale. FAST=face-arm-speech-time test.

**Figure 3:**
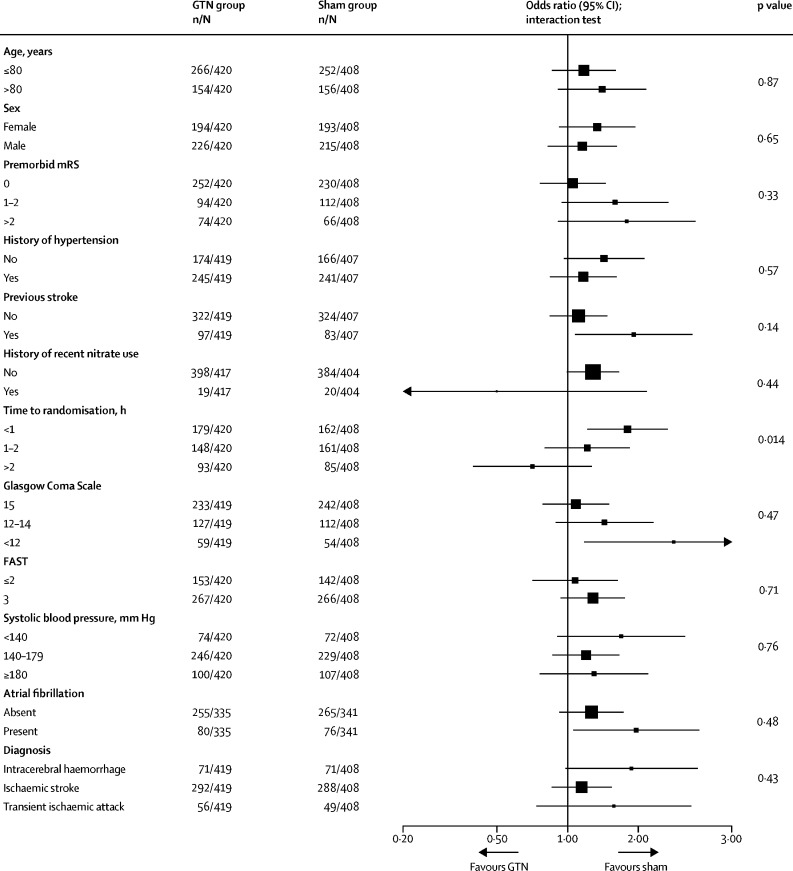
Effect of GTN versus sham on mRS score at day 90 in cohort 1 prespecified subgroups defined before treatment and admission to hospital Comparison by ordinal logistic regression adjusted for age, sex, premorbid mRS, FAST, pretreatment systolic blood pressure, index event (intracerebral haemorrhage, ischaemic stroke, transient ischaemic attack, mimic), time to randomisation, and reperfusion therapy (alteplase, intra-arterial therapy, none). GTN=glyceryl trinitrate. mRS=modified Rankin Scale. FAST=face-arm-speech-time test.

In the target disease population of cohort 1 (confirmed stroke and transient ischaemic attack), we found no strong evidence of an effect of GTN on functional outcome at 90 days compared with sham (mRS 3 [IQR 2–5] in the GTN group *vs* 3 [2–5] in the sham group; adjusted common OR [acOR] 1·25 [95 % CI 0·97–1·60]; p=0·083; table 2; figure 2), and the acOR of 1·25 suggests a tendency in favour of sham treatment. In sensitivity analyses, no difference was found in mRS when compared as mean difference, proportions with poor outcome (mRS >2), mRS in the per-protocol population, or when data were imputed for participants without a recorded mRS at day 90 (table 2). A significant interaction of the effect of GTN on mRS was present for time to randomisation, with a negative effect of GTN apparent in participants recruited within 1 h of symptom onset (figure 3); no other significant effect modification by subgroups was detected. Post-hoc assessment of the treatment effect on mRS in clinically relevant subgroups defined on or after admission to hospital (ie, potentially affected by treatment) showed a significant interaction with a worse outcome in patients with a more severe stroke on admission to hospital (post-treatment NIHSS >12; appendix).

When assessed in the target disease population (cohort 1), mRS did not differ between GTN and sham groups in participants with stroke (3 [IQR 2–6] in the GTN group *vs* 3 [2–5] in the sham group; acOR 1·26 [95% CI 0·96–1·64]; p=0·095; n=722), ischaemic stroke (3 [2–5] in GTN and sham groups; 1·15 [0·85–1·54]; p=0·36; n=580), or transient ischaemic attack (3 [1–3] in the GTN group *vs* 2 [1–3] in the sham group; 1·57 [0·74–3·35]; p=0·24; n=105). However, GTN was associated with a non-significantly worse outcome in patients with a final diagnosis of intracerebral haemorrhage (5 [4–6] in the GTN group *vs* 5 [3–5] in the sham group; 1·87 [0·98–3·57]; p=0·057; n=142; appendix).

Analysis of the ITT population (cohort 2) also showed that mRS did not differ between GTN and sham groups in the primary analysis (3 [IQR 2–5] for both groups; acOR 1·04 [95% CI 0·84–1·29]; p=0·69; table 2; appendix) or in any sensitivity analysis (data not shown). In predefined subgroups, a significant interaction was seen by final diagnosis (appendix); in contrast to the effect of GTN in stroke or transient ischaemic attack (see above), GTN appeared to be associated with an improved mRS in patients with a mimic (non-stroke or transient ischaemic attack mimic; 3 [1–4] for both groups; 0·54 [0·34–0·85]; p=0·0081); in a post-hoc analysis, this positive finding was not localised to any particular type of mimic (data not shown).

GCS at admission to hospital was 0·4 points lower in the GTN group. Because of this difference, we performed a post-hoc sensitivity analysis adding baseline GCS to the statistical model for the primary outcome in cohort 1, which had minimal effect on the result (OR 1·22 [95% CI 0·95–1·56]). Otherwise, we found no evidence of any other differences between GTN and sham groups in secondary outcomes in cohort 1 (table 2; appendix). A global analysis encompassing the primary outcome and prespecified secondary outcomes showed no difference (table 2; appendix).

Compared with the sham group, patients with an ischaemic stroke in the GTN group were less likely to have thrombectomy (appendix); conversely, patients in the GTN group were more likely to be ventilated in an intensive care unit. Use of other standard stroke treatments did not differ between the randomised treatment groups. No differences were found between GTN and sham groups in secondary outcomes at day 90 (table 2).

The proportion of deaths at day 4 did not differ between GTN and sham groups either in the target disease population (cohort 1) or in the full ITT population (cohort 2; table 2). Similarly, the proportion of deaths by day 90 did not differ between groups in cohort 1 (adjusted HR [aHR] 1·24 [95% CI 0·91–1·68]; p=0·17; table 2; appendix) or in cohort 2. The most common causes of death were progression or recurrence of the index stroke and pneumonia. A slight excess of headaches by day 4 was apparent in the GTN group. The number of participants experiencing one or more serious adverse events did not differ between the GTN and sham groups (188 [33%] patients *vs* 170 [29%]; p=0·16; appendix) although cardiovascular serious adverse events were more common in the GTN group (29% [5%] *vs* 16 [3%]). No suspected unexpected serious adverse reactions occurred.

The on-treatment hospital-based imaging findings for participants with a final diagnosis of stroke or transient ischaemic attack are shown in the appendix. In ischaemic stroke, scanning was done on admission at 2·2 h and on day 2 at 27·7 h after onset of stroke or transient ischaemic attack. No differences were found between GTN and sham groups in respect of infarct size, swelling, or mass effect on plain brain CT. Patients receiving intravenous thrombolysis were non-significantly less likely to have haemorrhagic transformation with GTN than with sham (5 [3%] *vs* 11 [8%]; OR 0·38 [95% CI 0·13–1·13]; p=0·082). For participants with intracerebral haemorrhage, scanning was done on admission at an average of 2·3 h and 28·9 h after onset of stroke or transient ischaemic attack (appendix). GTN was associated with larger haematoma than sham (1·95 [1·07–3·58]; p=0·030) and more mass effect (2·42 [1·26–4·68]; p=0·0083) at hospital admission.

Addition of the results for participants with confirmed stroke or transient ischaemic attack in cohort 1 (target disease population) in RIGHT-2 to the positive published Cochrane review^14^ for hyperacute administration of GTN, resulted in neutral effects for end-of-trial death or dependency (mRS >2; OR 0·80 [95% CI 0·59–1·10]; p=0·17; heterogeneity *I*^2^=16%; p=0·30) and death (0·52 [0·16–1·72]; p=0·28; *I*^2^=86%; p=0·0007; appendix). Heterogeneity between trial results was apparent for death, emphasising the difference between the results for RIGHT-2 versus the earlier RIGHT^10^ and ENOS-early^11^, ^12^ trials.

## Discussion

RIGHT-2 recruited 1149 patients who were taken to 54 hospitals; 516 paramedics from 184 ambulance stations within eight UK ambulance services performed screening, obtained consent, and delivered treatment and early follow-up measurement. Consent or proxy consent was obtained from patients, relatives, carers, or friends of the patient, or by the recruiting paramedic. Treatment was commenced very early, and faster than in hospital-based trials, with 38% of patients treated in the first 60 min after stroke onset (the so-called golden hour).^25^ Hence, we have shown that it is feasible to perform a large multicentre, paramedic-delivered, ambulance-based trial in patients with suspected stroke in the UK. Having shown feasibility, we compared the effect of GTN with sham and found that treatment with GTN did not affect functional outcome in patients with the target diagnosis of confirmed stroke or transient ischaemic attack or in the overall recruited population.

The results shown for GTN differ from those reported in a previous small phase 2, ambulance-based trial (RIGHT,^10^ with recruitment <4 h of onset) and a subgroup of a large phase 3 trial (ENOS,^12^ recruitment <6 h of onset). These discrepant results have several potential explanations. First, GTN might simply be ineffective in very early stroke, as suggested by the absence of any effect of GTN on multiple secondary outcomes and a global outcome and by neutral meta-analyses when combining ENOS-early, RIGHT, and RIGHT-2. Second, the discrepant findings might be due to chance rather than any true positive or negative effect of GTN. Chance could also account for the observation that GTN appeared to be beneficial in participants with a final diagnosis of non-stroke or transient ischaemic attack mimic irrespective of the underlying mimic diagnosis. Third, the difference between RIGHT-2 and ENOS-early or RIGHT might be real, due to intrinsic differences in their design: in RIGHT-2, we randomly assigned patients far earlier (median 71 min) than in RIGHT and ENOS-early combined (median 257 min) and so will have recruited a different cohort of patients. Compared with these earlier trials, participants in RIGHT-2 were older and more likely to have premorbid dependency, diabetes, previous stroke, and ischaemic heart disease; and, among the patients with intracerebral haemorrhage, they were more likely to still be in a period of haematoma expansion. All these factors might have contributed to different effects of GTN on functional outcome, as was apparent for reductions in systolic blood pressure (6·2 mm Hg in RIGHT-2 *vs* 9·4 mm Hg in ENOS-early).^11^, ^12^ Finally, studies showing a positive effect of GTN within 6 h used a 7-day treatment period and had higher rates of adherence, so it is conceivable that GTN was not given for long enough in RIGHT-2.

Although RIGHT-2 was a neutral trial, GTN was associated with a tendency for a worse functional outcome in patients with confirmed stroke or transient ischaemic attack (cohort 1), with a 95% CI covering a range from a clinically insignificant benefit (OR 0·97) to a clinically significant hazard (OR 1·60). This tendency towards harm was particularly seen in patients with intracerebral haemorrhage, very early stroke (<1 h), and severe stroke (GCS <12, NIHSS >12). Further, the imaging findings support a negative effect of GTN in ultra-acute intracerebral haemorrhage with larger haematoma, and more haematoma expansion, perihaematoma oedema, mass effect, and midline shift. There are several explanations for the potential hazard in intracerebral haemorrhage, which has a higher base rate of ultra-early neurological deterioration than ischaemic stroke,^26^ and these are given in decreasing order of likelihood. First, the earliest stage in haemostasis is vasoconstriction and GTN might prevent this protective response and so lead to very early haematoma expansion. Second, although we did not identify antiplatelet effects with GTN in a previous study of patients with stroke,^7^ others have reported this response in laboratory experiments,^27^ and GTN could therefore have amplified haematoma expansion in intracerebral haemorrhage thereby countering any effects of lowering blood pressure. Third, venodilators, such as sodium nitroprusside and GTN, have been shown experimentally and clinically to raise intracerebral pressure and reduce cerebral blood flow, particularly if intracerebral pressure is already elevated.^28^, ^29^ Reduced blood flow might then induce peri-haematoma ischaemia. Although pilot work did not find a negative effect of GTN on cerebral blood flow or cerebral perfusion pressure in patients in hospital with recent stroke,^8^, ^9^, ^30^ these studies were not in the ultra-acute period after stroke and were mainly in ischaemic stroke, and so they might not be directly relevant to the RIGHT-2 population. Last, GTN can stimulate the formation of reactive oxygen species such as superoxide (O_2_^−^) and peroxynitrite (OONO^−^), which might attenuate vasodilation and increase the potential for cellular damage.^31^

Preclinical studies of neuroprotective and collateral enhancement therapy in ischaemic stroke suggest that treatment is most effective when administered rapidly after symptom onset. Ideally treatment would be started before hospital admission to reduce stroke-to-needle time.^32^ The US FAST-MAG trial^15^ successfully randomly assigned 1700 participants in ambulances to receive intravenous magnesium or placebo within 2 h of symptom start and took them to 36 hospitals. RIGHT-2 extends these observations showing that a large stroke ambulance trial can be performed embedded in the UK national health service involving multiple ambulance services and hospitals. Hence, other interventions that do not require previous CT scanning could be tested in this environment in the future. By extrapolation, paramedics will also be able to administer such interventions routinely in the ambulance once they have been shown to be effective in one or more types of stroke and safe in mimics.

The present trial has several strengths, including the large sample size, generalisability due to wide inclusion criteria, central concealment of treatment assignment, excellent adherence to the first dose of allocated treatment, prospective collection of multiple functional outcomes and safety measures such as hypotension and hypertension, near-complete follow-up (96% of patients had their primary outcome recorded), and central masked assessment of outcomes at day 90. Patients received modern care, including stroke unit admission, thrombolysis, thrombectomy, and hemicraniectomy.

Several limitations are also present. First, GTN was administered in a single-blind design since no commercial sources of placebo patches are available. Despite this design, patient-blinding at day 90 was successful through use of a near identical sham patch, and both GTN and sham patches were unmarked; placement of a gauze dressing over the patch^8^, ^9^, ^10^ gave additional blinding. Further, outcomes measured at day 90 were assessed centrally by trained staff masked to treatment assignment who were not involved in hospital care of enrolled patients. Second, many patients did not receive randomised treatment for the intended minimum period of 2 days, and even fewer for the full 4 days; hence, participants might have received inadequate treatment. Third, the difference in blood pressure between GTN and sham was small and less than that seen in the large ENOS trial.^11^, ^12^ Although this difference might reflect inaccuracies in blood pressure measurement in the emergency environment of an ambulance and hospital admission, it might also explain the lack of benefit in ischaemic stroke. Fourth, we had to increase the sample size, an unplanned change that was necessary because of the unexpectedly high mimic rate. Last, the trial's wide inclusion criteria recruited a population of patients with stroke that would not normally enter hospital-based trials. In this respect, a group of participants with very severe intracerebral haemorrhage were enrolled who deteriorated rapidly and then died, which could have neutralised any treatment effect.

As far we are aware, RIGHT-2 is the first acute stroke trial to use a hierarchical approach to analysis in which the first analysis in the primary family was performed in the target population, with the potential for a subsequent primary analysis across the entire ITT population. We followed this predefined plan^17^ since the high mimic rate had the potential to dilute any treatment effect. Although the non-positive result in the target population precluded testing the ITT population, this approach had the advantage that the primary analysis of the study directly addressed the core question of the biological benefit of drug administration in patients with the disease of interest.

In summary, treatment with transdermal GTN administered before hospital did not alter functional outcome in participants with ultra-acute stroke. The signals of potential adverse effect of GTN in intracerebral haemorrhage are not definitive, but suggest the advisability of close safety monitoring in ongoing trials of prehospital GTN in ultra-acute stroke (ISRCTN99503308). Nevertheless, earlier findings in the large ENOS trial, including in intracerebral haemorrhage, suggest that transdermal GTN is safe when administered later in hospital^11^ and might continue to be used for lowering blood pressure, for example before thrombolysis. Finally, the study shows that large ambulance-based studies are feasible in the UK and, by extrapolation and taking into account the FAST-MAG trial,^15^ in most developed countries.

## Data sharing

Individual participant data will be shared with the Virtual International Stroke Trials Archive (VISTA) collaboration. From Jan 1, 2021, the Chief Investigator (with approval from the Trial Steering Committee as necessary) will consider other requests to share individual participant data via email at: right-2@nottingham.ac.uk. We will require a protocol detailing hypothesis, aims, analyses, and intended tables and figures. Where possible, we will perform the analyses; alternatively, de-identified data and a data dictionary will be supplied for the necessary variables for remote analysis. Any sharing will be subject to a signed data access agreement. Ultimately, the data will be published.
