# Supplementary material for: Prehospital transdermal glyceryl trinitrate in patients with ultra-acute presumed stroke (RIGHT-2): an ambulance-based, randomised, sham-controlled, blinded, phase 3 trial
Source: Lancet. 2019 Mar 9;393(10175):1009–20. doi: 10.1016/S0140-6736(19)30194-1 (PMC6497986; doi:10.1016/S0140-6736(19)30194-1)

THE LANCET

Supplementary appendix

This appendix formed part of the original submission and has been peer reviewed. We post it as supplied by the authors.

Supplement to: The RIGHT-2 Investigators. Prehospital transdermal glyceryl trinitrate in patients with ultra-acute presumed stroke (RIGHT-2): an ambulance-based, randomised, sham-controlled, blinded, phase 3 trial. *Lancet* 2019; published online Feb 6. http://dx.doi.org/10.1016/S0140-6736(19)30194-1.

APPENDIX

TABLE OF CONTENTS

	Page
Title	1
Study Investigators	1
Methods, additional information	5
Results, additional information	10
References, additional	12
List of supplementary Tables and Figures	14
Tables (start from)	15
Figures (start from)	24

Title

Pre-hospital transdermal glyceryl trinitrate in patients with ultra-acute presumed stroke (RIGHT-2): a randomised, sham-controlled, blinded, phase III, superiority ambulance-based trial

* Study Investigators

Writing Committee

Philip M Bath, Polly Scutt, Craig S Anderson, Sandeep Ankolekar, Jason P Appleton, Eivind Berge, Lesley Cala, Mark Dixon, Timothy J England, Peter J Godolphin, Diane Havard, Lee Haywood, Trish Hepburn, Kailash Krishnan, Grant Mair, Alan A Montgomery, Keith Muir, Stephen J Phillips, Stuart Pocock, John Potter, Chris Price, Marc Randall, Thompson G Robinson, Christine Roffe, Peter M Rothwell, Else C Sandset, Nerses Sanossian, Jeffrey L Saver, Angela Shone, A Niroshan Siriwardena, Joanna M Wardlaw, Lisa J Woodhouse, Graham Venables, Nikola Sprigg

Trial Steering Committee

Independent experts: Graham Venables (TSC Chair/Neurologist; Sheffield), Pierre Amarenco (Neurologist; Paris, France), Keith Muir (Neurologist; Glasgow)
Grant holders: Philip M Bath (Chief Investigator/Stroke Physician; Nottingham), Timothy J England (Stroke Physician; Derby), Nikola Sprigg (Stroke Physician; Nottingham) , Alan A Montgomery (Statistician; Nottingham), Stuart Pocock (Statistician; London), John Potter (Stroke Physician; Norwich), Chris Price (Stroke Physician; Newcastle), Thompson G Robinson (Stroke Physician; Leicester), Christine Roffe (Stroke Physician; Stoke-on-Trent), Niroshan Siriwardena (Pre-Hospital Healthcare; Lincoln), Joanna Wardlaw (Neuroradiologist; Edinburgh)
Observers (Nottingham): Polly Scutt (Statistician), Jason P Appleton (Medic), Mark Dixon (Paramedic), Lisa J Woodhouse (Statistician)
Funder's representative: Shannon Amoils (London)
Patient representative: Malcolm Jarvis (Nottingham)
Sponsor's representative: Angela Shone (Nottingham)
Observers (Nottingham): Jason Appleton (Medic), Mark Dixon (Paramedic), Polly Scutt (Statistician), Lisa J Woodhouse (Statistician)

International Advisory Committee

Craig C Anderson (Sydney, Australia), Eivind Berge (Oslo, Norway), Peter M Rothwell (Oxford, UK), Stephen J Phillips (Halifax, Canada), Else Sandset (Oslo, Norway), Nerses Sanossian (Los Angeles, USA), Jeff L Saver (Los Angeles, USA)

Independent Data Monitoring Committee
Peter Sandercock (Chair; Edinburgh, UK): Kjell Asplund (Umeå, Sweden), Colin Baigent (Oxford, UK)

Independent Events (SAE) Adjudicators
Marc Randall (Leeds), Sandeep Ankolekar (London)

Neuroimaging Adjudicators
Joanna Wardlaw (Chair; Edinburgh, UK), Lesley Cala (Perth, Australia), Grant Mair (Edinburgh, UK)

Trial Management Committee (Nottingham)
Senior Trial Manager/Chair: Diane Havard
Chief Investigator: Philip Bath
National Paramedic: Mark Dixon
Senior Trial Coordinator: Harriet Howard
Outcome Coordinators: Christopher Lysons (-06/18), Gemma Walker (-03/16), Hayley Gregory (-01/18), James Kirby (-12/16), Jennifer Smithson (-06/16), Joanne Keeling (-09/16), Nadia Frowd (-06/18), Robert Gray
Statisticians: Polly Scutt, Lisa Woodhouse
Programming/database management: Lee Haywood, Richard Dooley
Finance: Wim Clarke (-08/17)
Secretary: Patricia Robinson
Physicians: Jason Appleton, Zhe Kang Law
Neuroimaging assessment: Joanna Wardlaw
Haematoma volume assessment: Kailash Krishnan

Pharmacy (Nottingham University Hospitals NHS Trust)
Sheila Hodgson (Nottingham), Adam Millington (Nottingham)

Neuroimaging management
Eleni Sakka, (Edinburgh, UK)

Programming/database management
Website/database (Nottingham, UK): Lee Haywood, Richard Dooley
Imaging (Edinburgh, UK): David Buchanan, Jeb Palmer

Participating Ambulance Services, Hospital Trusts and Investigators (with recruitment totals)

East Midlands
- East Midlands Ambulance Service NHS Trust (218): AN Siriwardena, D Shaw, H Cobb, R Johnson, T Payne, R Spaight, A Spaight
- Chesterfield Royal Hospital (21): M A Sajid, A Whileman, E Hall, H Cripps, J Toms, R Gascoyne, S Wright
- King's Mill Hospital (23): M Cooper, A Palfreman, A Rajapakse, I Wynter
- Leicester Royal Infirmary (26): K Musarrat, A Mistri, C Patel, C Stephens, S Khan, S Patras

- Lincoln County Hospital (39): M Soliman, A Elmarimi, C Hewitt, E Watson, I Wahishi, J Hindle, L Perkin, M Wills, S Arif, S Leach, S Butler
- Northampton General Hospital (10): D O'Kane, C Smith, J O'Callaghan
- Nottingham City Hospital (38): W Sunman, A Buck, B Jackson, C Richardson, G Wilkes, J Clarke, L Ryan, O Matias
- Pilgrim Hospital (5): D Mangion, A Hardwick, C Constantin, I Thomas, K Netherton, S Markova
- Royal Derby Hospital (33): T England, A Hedstrom, B Rushton, C Hyde, J Scott, M Blair, M Maddula, R Donnelly, S Keane. S Johnson, H McKenzie
- Scunthorpe General Hospital (11): A Banerjee, D Hutchinson, H Goodhand, J Hill, K Mellows, M Cheeseman, V McTaggart

East of England
- East of England Ambulance Service NHS Trust (178): T Foster, L Prothero
- Colchester General Hospital (59): R Saksena, A O'Kelly
- James Paget Hospital (19): H Wyllie, C Hacon, H Nutt, J North, K Goffin
- Norfolk and Norwich University Hospital (23): J Potter, A Wiltshire, G Ravenhill, K Metcalf, L Ford, M Langley, W Davison
- Peterborough City Hospital (52): S Subramonian, F Magezi, I Obi, N Temple, N Butterworth-Cowin, P Owusu-Agyei
- West Suffolk Hospital (31): A F M Azim, A Nicolson, J Imam, J White, L Wood

London
- London Ambulance Service NHS Trust (202): R Fothergill, N Thomson, J Lazarus, H Werts
- King's College Hospital (50): L Sztriha, C Ho, E McKenzie, E Owoyele, J Lim, J Aeron-Thomas, M Docksey, N Sylvester, P Rao
- The Royal London Hospital (64): B M Bloom, E Erumere, G Norman, I Skene, L Cuenoud, L Howaniec, O Boulton, P Daboo, R Michael, S Al-Saadi, T Harrison, H Syed, L Argandona, S Amiani
- University College Hospital (86): R Perry, A Ashton, A Banaras, C Hogan, C Watchurst, E Elliott, N Francia, N Oji, R Erande, S Obarey, S Feerick, S Tshuma

South Central
- South Central Ambulance Service NHS Foundation Trust (7): E England, H Pocock, K Poole
- Wycombe Hospital (7): S Manchanda, I Burn, S Dayal

South West
- South Western Ambulance Service NHS Trust (264): K McNee, M Robinson, R Hancock, A South
- Bristol Royal Infirmary (17): C Holmes, A Steele, L B Guthrie, M Osborn
- Derriford Hospital (27): A Mohd Nor, B Hyams, C Eglinton, D Waugh, E Cann, N Wilmshurst, S Piesley, S Shave
- Gloucestershire Royal Hospital (31): D Dutta, M Obeid, D Ward, J Turfrey, J Glass, K Bowstead, L Hill, P Brown, S Beames, S O'Connell, V Hughes
- Musgrove Park Hospital (7): R Whiting, J Gagg, M Hussain, M Harvey, D Karunatilake, B Pusuluri, A Whitcher, C Pawley, J Allen, J Foot, J Rowe, C Lane
- Poole Hospital (10): S Ragab, B Wadams, J Dube
- Royal Bournemouth Hospital (11): B Jupp, A Ljubez, C Bagnall, G Hann, L Tucker, M Keltos, S Orr
- Royal Cornwall Hospital (31): F Harrington, A James, A Lydon, G Courtauld, K Bond, L Lucas, T Nisbett

- Royal Devon and Exeter Hospital (26): J Kubie, A Bowring, G Jennings, K Thorpe, N Mason, S Keenan
- Royal United Hospital Bath (38): L Gbadamoshi, D Howcroft, H Newton, J Choulerton, J Avis, L Shaw, P Paterson, P Kaye, S Hierons, S Lucas
- Southmead Hospital (15): P Clatworthy, B Faulkner, L Rannigan, R Worner
- Torbay Hospital (13): B Bhaskaran, A Saulat, H Bearne, J Garfield-Smith, K Horan, P Fitzell, S Szabo
- Weston General Hospital: M Haley, D Simmons, D Cotterill, G Saunders, H Dymond, S Beech
- Yeovil District Hospital (37): K Rashed, A Tanate, C Buckley, D Wood, L Matthews, S Board, T Pitt-Kerby

Wales
- Welsh Ambulance Service NHS Trust (87): N Rees, C Convery
- Bronglais General Hospital (32): P Jones, C Bryant, H Tench, M Dixon, R Loosley, S Coetzee, S Jones, T Sims
- Morriston Hospital (27): M Krishnan, C Davies, L Quinn, L Connor, M Wani, S Storton, S Treadwell, T Anjum
- Royal Gwent Hospital (25): C Somashekar, A Chandler, C Triscott, L Bevan, M Sander, S Buckle
- Wrexham Maelor Hospital (2): W Sayed, K Andrews, L Hughes, R Hughes

West Midlands
- West Midlands Ambulance Service NHS Foundation Trust (37): M Ward, A Pretty, A Rosser, B Davidson, G Price, I Gunson, J Lumley-Holmes, J Miller, M Larden, M Jhamat, P Horwood, R Boldy
- Hereford County Hospital (3): C Jenkins, F Price, M Harrison, T Martin
- New Cross Hospital (16): N Ahmad, A Willberry, A Stevens, K Fotherby
- Royal Stoke University Hospital (3): C Roffe, A Barry, A Remegoso, F Alipio, H Maguire, J Hiden, K Finney, R Varquez
- Sandwell General Hospital (9): S Ispoglou, A Hayes, D Gull, R Evans
- Walsall Manor Hospital (5): E Epstein, S Hurdowar

Yorkshire
- Yorkshire Ambulance Service NHS Trust (153): J Crossley, J Miles, K Hird, R Pilbery
- Bradford Royal Infirmary (3): C Patterson, H Ramadan, K Stewart, O Quinn, R Bellfield, S Maguire, W Gaba
- Calderdale Royal Hospital (26): A Nair, A Wilson, C Hawksworth, I Alam, J Greig, M Robinson, P Gomes, P Rana, Z Ahmed
- Doncaster Royal Infirmary (13): P Anderton, A Neal, D Walstow, R Fong
- Harrogate District Hospital (4): S Brotheridge, A Bwalya, A Gillespie, C Midgley, C Hare, H Lyon, L Stephenson, M Broome, R Worton, S Jackson
- Hull Royal Infirmary (27): R Rayessa, A Abdul-Hamid, C Naylor, E Clarkson
- Leeds General Infirmary (31): A Hassan, D Waugh, E Veraque, L Finch, L Makawa
- Pinderfields General Hospital (14): M Carpenter, P Datta, A Needle, L Jackson, HJ Brooke, J Ball, T Lowry
- Rotherham General Hospital (4): S Punnoose, R Walker, V Murray
- Royal Hallamshire Hospital (12): A Ali, C Kamara, C Doyle, E Richards, J Howe, K Dakin, K Harkness, R Lindert
- York Hospital (25): P Wanklyn, P Willcoxson, P Clark Brown, R Mir

Note: Ambulance Service patient totals may not equal sum of hospital totals because of overlapping of AS regional coverage

METHODS, ADDITIONAL INFORMATION

Training of Investigators

Paramedics
RIGHT-2 paramedics were trained in the protocol, and those parts of Good Clinical Practice that are relevant to their research role in RIGHT-2. Training was provided by video and followed by a pass/fail multiple choice questionnaire. A training manual supported this material.
Video: http://right-2.ac.uk/?ZVideo
Training manual: http://right-2.ac.uk/docs/ptplan10

Hospital-based Investigators
RIGHT-2 hospital-based investigators with current Good Clinical Practice were trained in the protocol and use of the National Institutes of Health Stroke Scale (NIHSS), modified Rankin Scale (mRS) and Barthel Index.

Day 90 outcome assessors
Outcome assessors were trained in, and then tested with case scenarios, for the mRS.

Schedule for Monitoring of Sites and Data Integrity

Site monitoring was performed by the Coordinating Centre at the Nottingham Stroke Trials Unit with the aim of ensuring quality control for the delivery of the protocol, collection of data, and adherence with UK regulations and ethics. Each recruiting Ambulance Service and Hospital site had a site initiation visit performed via video for training, and then at least one monitoring visit performed after 5 patients had been enrolled; further visits were performed as deemed necessary. Monitoring visits confirmed the presence of participants and their consent, eligibility criteria, selected data critical to the trial (core demographics, prescription of interventions, and blood pressure), and reported serious adverse events.

Central statistical monitoring of the data was performed according to Buyse *et al* [1] during the trial and prior to final locking of the data. Checks included logic and range checks, digit preference, comparison of univariate data between sites, and comparison of multiple variable models between countries. The monitoring procedures were compliant with the requirements of the sponsor, the national ethics committee and regulatory authority, and fulfilled Good Clinical Practice requirements.

Sample size and justification

The null hypothesis (H_0) is that GTN will not shift the mRS in participants with ultra-acute stroke. The alternative hypothesis (H_1) is that mRS will shift the mRS between those stroke participants randomised to GTN versus sham (2-sided). In calculating the sample size, treatment was assumed to have similar efficacy in IS and ICH,[2] and to have a neutral effect in transient ischaemic attack (TIA) and stroke mimics. The shift in outcome distribution of the modified Rankin Scale was projected to be approximately 25% of the effect observed in the RIGHT pilot trial of glyceryl trinitrate.[3] A total sample size of 850 participants (425 in each arm) was required to detect a shift in mRS with a common odds ratio of 0·70 (equivalent to a binary odds ratio of 0·66). This assumed an overall significance level (alpha, type I error) of 5%,

and 90% power (1-beta, type II error), distribution of mRS scores (as shown below), mimic and TIA rate of 20%, 3% loss to follow-up, and reduction for baseline co-variate adjustment of 20%.[4]

Distribution of mRS from [5]

0	1	2	3	4	5	Dead/6
2%	17%	20%	15%	10%	12%	24%

During the trial, blinded analysis revealed that the mimic rate was higher than expected at ~25% with TIA at ~10%, i.e. the rate of expected non-responders was more than one-third of participants. Funding, ethics and regulatory approval was sought for an increase in sample size to 1050 (rounded up from 1015) and this was approved. A decision to perform a hierarchical analysis was made following concerns that the high mimic rate would neutralise any significant treatment effect; this decision was made blinded to any between-group comparisons of outcome data, and was published in the statistical analysis plan.[6] The sequential analysis would be performed in two prespecified, progressively inclusive cohorts based on the final in-hospital diagnosis: stroke or TIA (cohort 1); and stroke, TIA, or non-stroke/TIA (mimic), i.e. all patients (cohort 2). TIA was included with stroke since GTN might move participants between a diagnosis of mild stroke and TIA;[6] further, it is not possible currently to distinguish stroke from TIA in the pre-hospital environment, and analysis of stroke and TIA together matches that done in previous hyperacute trials where some TIAs are inevitably included.

Inclusion/exclusion criteria (from protocol)

Inclusion criteria

- Patients presenting to paramedics in context of 999 ambulance call for 'stroke'.
- Age 18 years or more (there is no maximum age).
- 'Face/Arm/Speech' Time (FAST) score 2 or more.
- Onset <=4 hours.
- Systolic BP >=120 mmHg.
- Have provided informed consent, or a relative/paramedic has provided proxy consent.
- Paramedic is trained in RIGHT-2 procedures, is from a participating ambulance station, and will take patient to a participating comprehensive/primary stroke centre.

Exclusion criteria

- Patient at a Nursing Home.
- Glucose (BM stix) <2.5 mmol/l.
- Glasgow Coma Scale <8.
- Witnessed seizure/fit at presentation.
- Known life expectancy <6 months.
- Known to have taken a PDE5 inhibitor, such as sildenafil, in previous day before stroke.
- Known sensitivity to Transiderm Nitro patch.
- Known sensitivity to Duoderm hydrocolloid dressing.
- Known previous enrolment into RIGHT-2.

Baseline assessments

Pre-randomisation
Paramedics recorded the following information in the field: age, sex, time of onset and randomisation, systolic and diastolic blood pressure, heart rate, Glasgow coma scale (GCS), face-arm-speech-time (FAST) score and ECG evidence of atrial fibrillation.[7]

Hospital arrival, non-modifiable
On arrival at hospital, staff recorded non-modifiable information that could not be altered by treatment: ethnic group, pre-morbid mRS, and medical history (hypertension, diabetes, previous stroke, ischaemic heart disease, and smoking).

Hospital arrival, modifiable
Hospital staff also recorded information that could have been modified by treatment: qualifying event (ICH, IS, TIA, mimic; e.g. treatment might move the diagnosis between IS and TIA), OCSP syndrome,[8] severity/impairment as NIHSS, and Glasgow coma scale. FAST was calculated from NIHSS. GCS and NIHSS were analysed as intermediate outcomes.

Procedures

Non-trial concomitant therapy followed UK national stroke practice guidelines.[9] We recommended that patients with a stroke should be admitted to a stroke unit, either directly or via the accident & emergency department. Patients with IS should receive, as appropriate, reperfusion therapy (intravenous alteplase and/or thrombectomy with an approved device), aspirin, and hemicraniectomy. Lowering BP was allowed, typically with labetalol, either to facilitate thrombolysis if necessary or to treat sustained severe hypertension (SBP >220 mmHg) in IS, and to intensively lower BP in ICH.

Definition of Events

See Statistical Analysis Plan.[6]

Neuroimaging Scan Adjudication

A plain CT scan (or MRI brain scan), with or without a CT angiogram, were performed on admission to hospital, according to local site practice. Sites were asked (but not mandated) to perform a follow-up research scan at day 2. Sites could perform follow-up scans at any time point after enrolment according to clinical need. All these neuroimages were submitted to the Neuroimaging Coordinating Centre in Edinburgh using one of two methods:

1. Uploaded onto the trial website as uncompressed encrypted non-anonymised digital DICOM files. Once the trial system had validated the files against the expected patient details, the files were then anonymised. Or,
2. Sent by courier on a CD-ROM or DVD, with files in DICOM format with pseudo-anonymisation of patient details; patients were identified by their unique study number and initials.

When reviewed, some images were in non-DICOM format (e.g. .PNG, .JPG) and these were converted to DICOM. The anonymised image files were presented to a panel of adjudicators using a browser-based system driven from the trial database. Adjudicators were trained and assessed using the ACCESS system

(www.sirs2/neuroimage.co.uk),[10,11] and reviewed scans blinded to treatment assignment. Adjudication parameters were derived from the IST-3 image adjudication system,[12] and included information on:

- Presence of an acute stroke lesion: location, extent, degree of tissue hypoattenuation if ischaemic, mass effect including midline shift, hyperattenuated artery, haemorrhagic transformation; and presence of secondary acute ischaemia; in addition, lobar location, extension to subarachnoid space or ventricles, and maximum dimensions in three orthogonal planes were recorded for ICH.
- Presence of pre-stroke changes: atrophy, white matter hyperintensities, old stroke lesions.

CT/MR angiograms were adjudicated according to the validated approach used in the Third International Stroke Trial (IST-3).[13,14]

Information from adjudication was used to inform the final diagnosis for all patients with a received scan; where clinical and radiological information were incongruent, JMW performed a second adjudication to confirm imaging findings. For the purposes of the RIGHT-2 trial, participants with a final diagnosis of primary subarachnoid haemorrhage were considered as non-stroke/TIA, i.e. they were analysed as part of the mimic group.

Statistical analysis

Since outcomes such as modified Rankin Scale, EQ-5D health status utility values (HSUV) and Barthel Index include scores for death (6, 0 and -5 respectively), and in case treatment was associated with asymmetric effects on death and other outcome measures (e.g. more death and less impairment), an extreme value for death was added to the other outcome scales:[15] NIHSS = 43, EQ-5D HSUV = 0, EQ-VAS = -1, t-MMSE = -1, TICS-M = -1, animal naming = -1, ZDS = 102.5, home time = -1, GCS = 2.

The per protocol group were defined as participants with all of the following: consent or proxy consent, randomisation within 4 hours of onset of symptoms, FAST score 2 or 3, systolic blood pressure $\geq$120 mmHg, Glasgow coma scale $\geq$8/15, glucose $\geq$2.5 mmol/l, not from a nursing home, received the first two GTN patches/sham dressings, and not in another trial.

The primary and secondary analyses were adjusted for key baseline prognostic variables to deal with any imbalances (whether obvious or not), and to increase statistical power.[4,6] Additionally, these analyses were adjusted for in-hospital reperfusion therapy as per the Statistical Analysis Plan.[6] Although treatment with GTN vs sham might influence rates of reperfusion therapy, the decision to include thrombolysis-mechanical thrombectomy in statistical adjustment was made on the basis that these therapies have profound positive effects on the modified Rankin scale and any imbalance between the treatment groups might alter the main trial findings.

Data Monitoring Committee (DMC)

The DMC was responsible for safeguarding the interests of trial patients, assessing the safety and efficacy of the intervention during the trial, assessing data integrity, and for monitoring the overall conduct of the trial. The DMC reviewed the recruitment of patients and assessed dichotomous safety and efficacy measures by treatment group.

Data were reviewed annually throughout the recruitment period of the trial; the Chair also reviewed data at 6-monthly intervals. The DMC was chartered to inform the Trial Steering Committee if, at any time, the results showed evidence beyond reasonable doubt of a difference between the randomised groups in the primary outcome. They also considered these data in the light of external information such as results from other completed trials. However, the DMC could perform statistical comparisons as they deemed necessary, with stopping criteria based on the Haybittle-Peto stopping rule (i.e. a difference of 3 standard errors is considered as clear evidence of a treatment effect).

The DMC were given specific criteria when considering recommendations to continue, alter or stop the trial:

For potential hazard

- Poor outcome (dichotomous analysis of the primary outcome, modified Rankin Scale >2) is less frequent in the sham/control group, P<0·01 (nominal, 2-sided) OR
- Death is less frequent in the sham/control group, P<0·01 (nominal, 2-sided).

For potential benefit

- Poor outcome (dichotomous analysis of the primary outcome, modified Rankin Scale >2) is less frequent in the GTN/active group, P<0·01 (nominal, 2-sided) AND
- Death is less frequent in the GTN/active group, P<0·01 (nominal, 2-sided) AND
- Poor outcome (modified Rankin Scale >2) is less frequent in the GTN/active group in ischaemic stroke, P<0·01 AND
- Poor outcome (modified Rankin Scale >2) in intracerebral haemorrhage, P<0·05 (nominal, 2-sided).

In making any decision, the DMC considered the overall internal and external evidence, and the need for a result that will persuade the medical scientific community bearing in mind the perceived simple and inexpensive nature of the treatment (in comparison with existing proven interventions such as intravenous thrombolysis, thrombectomy, hemicraniectomy and stroke unit care). The study was not terminated early.

RESULTS, ADDITIONAL INFORMATION

Protocol amendments

Five protocol amendments were made during the trial, these covering a variety of topics. Three key changes were made (in order of introduction):[16]

1. Addition of a substudy exploring the experiences and perspectives of participating paramedics. This substudy will be published separately
2. Increase in sample size from 850 [17] to 1050 and then 1100 patients reflecting the presence of a higher than anticipated proportion of mimics (i.e. not stroke or TIA, originally expected to be ~12% but in reality reaching almost 25%)
3. Change from a single intention-to-treat analysis involving all patients to a hierarchical analysis with the first in the target population of patients with a final diagnosis of stroke or TIA (i.e. excluding mimics), and the second in all patients.[6] Again, this reflected the presence of a higher than anticipated proportion of mimics and the expectation that these would dilute out any potential treatment effect

All protocol amendments were made blinded to treatment assignment and prior to database lock.

Sample size

The original required sample size of 850 (Methods, Statistical analysis) was increased to 1050 when blinded analysis revealed recruitment of a higher-than-expected rate of mimics; funding, ethics and regulatory approval supported this increase. Recruitment of 1050 participants was achieved in early March 2018 and since there was remaining funding left, it was agreed by the Sponsor and Trial management Committee to continue recruitment to the planned end in late May 2018 (appendix Table S1); hence, a total of 1149 participants were recruited.

Imaging outcomes

Appendix Table 7 gives selected imaging characteristics for both the hospital-admission and day 2-4 scans.

Ischaemic stroke

The time from onset to scanning slightly exceeded 2 hours in both treatment groups (appendix Table 7). Most infarcts were in the middle cerebral artery territory (data not shown). Infarct size and old changes (atrophy, peri-ventricular lucencies, old stroke) did not differ between the GTN and sham groups.

Intracerebral haemorrhage

Scanning was performed, on average, 2.3 hours after ictus (appendix Table 7). There were non-significant tendencies for haematoma to be larger on baseline scanning in the GTN group, and for more to expand. GTN treatment was associated with more mass effect at hospital admission, and a greater midline shift on day 2-4.

Planned secondary analyses, systematic reviews/meta-analyses and data sharing

Secondary analyses are planned (updated from [6]):

- Effect of GTN in ICH
- Effect of GTN in IS
- Effect of GTN in TIA

- Effect of GTN in mimics
- Effect of GTN on haemodynamics, and influence of hydration status
- Description of mimics and outcomes
- Logistics of ambulance recruitment
- Paramedic consent and management of a trial investigational medicinal product
- Paramedic views on participating in the trial
- Update of earlier ENOS secondary publications
- Integrated analysis of RIGHT-2, with ENOS, TARDIS and TICH-2 [15,18,19] for dependency, disability, cognition, mood and quality of life

Summary data will be used to update Cochrane collaboration systematic reviews and meta-analyses:

1. Nitric oxide donors (nitrates), L-arginine, or nitric oxide synthase inhibitors for acute stroke [20]
2. Interventions for deliberately altering blood pressure in acute stroke [21]

Individual participant data (IPD) will be used to update and perform new systematic reviews and meta-analyses as part of the Blood pressure in Acute Stroke Collaboration (BASC):[22,23]

1. Glyceryl trinitrate for acute stroke [2]
2. Lowering blood pressure for acute intracerebral haemorrhage
3. Lowering blood pressure for acute ischaemic stroke
4. Lowering blood pressure for acute ischaemic stroke with perperfusion

Individual participant data will be shared with the international (VISTA) Collaboration.[24,25] From, 1/1/2021, the Chief Investigator (with TSC approval as necessary) will consider other requests to share IPD via email at: right-2@nottingham.ac.uk. We will require a protocol detailing hypothesis, aims, analyses and intended tables and figures. Where possible, we will perform the analyses; alternatively, deidentified data and a data dictionary will be supplied for the necessary variables for remote analysis. Any sharing will be subject to a signed data access agreement. Ultimately, the data will be published.[26]

REFERENCES

1. Buyse M, George SL, Evans S, et al. The role of biostatistics in the prevention, detection and treatment of fraud in clinical trials. *Stat Med* 1999; **18**(24): 3435-51.
2. Bath P, Woodhouse L, Krishnan K, et al. Effect of treatment delay, stroke type, and thrombolysis on the effect of glyceryl trinitrate, a nitric oxide donor, on outcome after acute stroke: a systematic review and meta-analysis of individual patient from randomised trials. *Stroke Research and Treatment* 2016; **2016:9706720**.
3. Ankolekar S, Fuller M, Cross I, et al. Feasibility of an Ambulance-Based Stroke Trial, and Safety of Glyceryl Trinitrate in Ultra-Acute Stroke The Rapid Intervention With Glyceryl Trinitrate in Hypertensive Stroke Trial (RIGHT, ISRCTN66434824). *Stroke* 2013; **44**(11): 3120-8.
4. Gray LJ, Bath PM, Collier T. Should stroke trials adjust functional outcome for baseline prognostic factors? *Stroke* 2009; **40**(3): 888-94.
5. Ankolekar S, Fuller M, Cross I, et al. Feasibility of an ambulance-based stroke trial, and safety of glyceryl trinitrate in ultra-acute stroke: the rapid intervention with glyceryl trinitrate in Hypertensive Stroke Trial (RIGHT, ISRCTN66434824). *Stroke* 2013; **44**(11): 3120-8.
6. Scutt P, Appleton JP, Dixon M, et al. Statistical analysis plan for the 'Rapid Intervention with Glyceryl trinitrate in Hypertensive stroke Trial-2 (RIGHT-2). *European Stroke Journal* 2018.
7. Nor A, McAllister C, Louw S, et al. Agreement between ambulance paramedic- and physician-recorded neurological signs with Face Arm Speech Test (FAST) in acute stroke patients. *Stroke* 2004; **35**(6): 1355-9.
8. Bamford J, Sandercock P, Dennis M, Burn J, Warlow C. Classification and natural history of clinically identifiable subtypes of cerebral infarction. *Lancet* 1991; **337**(8756): 1521-6.
9. Intercollegiate Stroke Working Party. National clinical guideline for stroke. *Royal College of Physicians* 2016; **5th Edition 2016**.
10. Wardlaw JM, Farrall AJ, Perry D, et al. Factors influencing the detection of early CT signs of cerebral ischemia: an internet-based, international multiobserver study. *Stroke* 2007; **38**(4): 1250-6.
11. Wardlaw JM, von Kummer R, Farrall AJ, Chappell FM, Hill M, Perry D. A large web-based observer reliability study of early ischaemic signs on computed tomography. The Acute Cerebral CT Evaluation of Stroke Study (ACCESS). *PLoS One* 2010; **5**(12): e15757.
12. IST-3 collaborative group. Association between brain imaging signs, early and late outcomes, and response to intravenous alteplase after acute ischaemic stroke in the third International Stroke Trial (IST-3): secondary analysis of a randomised controlled trial. *The Lancet Neurology* 2015; **14**(5): 485-96.
13. Wardlaw JM, von Kummer R, Carpenter T, et al. Protocol for the perfusion and angiography imaging sub-study of the Third International Stroke Trial (IST-3) of alteplase treatment within six-hours of acute ischemic stroke. *Int J Stroke* 2015; **10**(6): 956-68.
14. Mair G, von Kummer R, Morris Z, et al. Effect of alteplase on the CT hyperdense artery sign and outcome after ischemic stroke. *Neurology* 2016; **86**(2): 118-25.
15. Bath PM, Woodhouse L, Scutt P, et al. Efficacy of nitric oxide, with or without continuing antihypertensive treatment, for management of high blood pressure in acute stroke (ENOS): a partial-factorial randomised controlled trial. *Lancet* 2015; **385**(9968): 617-28.
16. Bath PM, Scutt P, Appleton JP, et al. Baseline characteristics of the 1149 patients recruited into the Rapid Intervention with Glyceryl trinitrate in Hypertensive

stroke Trial-2 (RIGHT-2) randomised controlled trial. *International Journal Stroke* 2018; **In press**.
17. Appleton JP, Scutt P, Dixon M, et al. Ambulance-delivered transdermal glyceryl trinitrate versus sham for ultra-acute stroke: Rationale, design and protocol for the Rapid Intervention with Glyceryl trinitrate in Hypertensive stroke Trial-2 (RIGHT-2) trial (ISRCTN26986053). *Int J Stroke* 2017: 1747493017724627.
18. Bath PM, Woodhouse LJ, Appleton JP, et al. Antiplatelet therapy with aspirin, clopidogrel, and dipyridamole versus clopidogrel alone or aspirin and dipyridamole in patients with acute cerebral ischaemia (TARDIS): a randomised, open-label, phase 3 superiority trial. *Lancet* 2018; **391**(10123): 850-9.
19. Sprigg N, Flaherty K, Appleton JP, et al. Tranexamic acid for hyperacute primary IntraCerebral Haemorrhage (TICH-2): an international randomised, placebo-controlled, phase 3 superiority trial. *Lancet* 2018; **391**(10135): 2107-15.
20. Bath PM, Krishnan K, Appleton JP. Nitric oxide donors (nitrates), L-arginine, or nitric oxide synthase inhibitors for acute stroke. *Cochrane Database Syst Rev* 2017; **4**: Cd000398.
21. Bath PM, Krishnan K. Interventions for deliberately altering blood pressure in acute stroke. *Cochrane Database Syst Rev* 2014; (10): Cd000039.
22. Bath FJ, Bath PMW. What is the correct management of blood pressure in acute stroke? The Blood pressure in Acute Stroke Collaboration. *Cerebrovascular diseases (Basel, Switzerland)* 1997; **7**: 205-13.
23. Sandset EC, Sanossian N, Woodhouse LJ, et al. Protocol for a prospective collaborative systematic review and meta-analysis of individual patient data from randomized controlled trials of vasoactive drugs in acute stroke: The Blood pressure in Acute Stroke Collaboration, stage-3. *Int J Stroke* 2018: 1747493018772733.
24. Ali M, Bath PM, Curram J, et al. The Virtual International Stroke Trials Archive. *Stroke* 2007; **38**(6): 1905-10.
25. Ali M, Bath P, Brady M, et al. Development, expansion, and use of a stroke clinical trials resource for novel exploratory analyses. *Int J Stroke* 2012; **7**(2): 133-8.
26. Sandercock PA, Niewada M, Czlonkowska A. The International Stroke Trial database. *Trials* 2011; **12**(1): 101.
27. Sandercock P, Wardlaw JM, Lindley RI, et al. The benefits and harms of intravenous thrombolysis with recombinant tissue plasminogen activator within 6 h of acute ischaemic stroke (the third international stroke trial [IST-3]): a randomised controlled trial. *Lancet* 2012; **379**(9834): 2352-63.

LIST OF SUPPLEMENTARY TABLES AND FIGURES

Tables		Page
1.	Trial timelines for trial delivery	15
2.	Adherence and reasons for non-adherence, all	16
3.	Blinding to treatment at day 90 follow-up	17
4.	Additional pre-specified outcome analyses, confirmed stroke/TIA patients	18
5.	In hospital management and treatment, confirmed stroke/TIA patients	20
6.	Serious adverse events, all patients	21
7.	Imaging, confirmed stroke/TIA patients	22
8.	Protocol violations, all patients	24

Figures		
1.	Consort flow diagram, confirmed stroke/TIA patients	25
2.	Systolic and diastolic blood pressure, confirmed stroke/TIA patients	26
3.	Systolic and diastolic blood pressure, all patients	27
4.	Distribution of mRS, all patients	28
5.	Forest plot of mRS in pre-specified subgroups, all patients	29
6.	Forest plot of mRS in *post hoc* subgroups, confirmed stroke/TIA patients	30
7.	Forest plot of global outcome components (Wei-Lachin test)	31
8.	Kaplan-Meier curve for death, confirmed stroke/TIA patients	32
9.	Kaplan-Meier curve for death, all patients	33
10.	Meta-analysis of effect of treatment on mRS, and death, confirmed stroke/TIA patients	34

Table 1. Trial timelines for trial delivery.

Event	Date	Notes
Outline grant application submitted	20/12/13	British Heart Foundation (BHF)
Invitation to submit full application	02/01/14	BHF
Grant application submitted	10/04/14	BHF
Funding approved	11/09/14	CS/14/4/30972
Protocol version 1·0	09/01/15	First version
Ethics approval	24/02/15	15/EM/0055
MHRA approval	01/03/15	03057/0064/001-0001
First patient recruited	22/10/15	
Protocol version 2·0	27/10/15	Clarified aspects, and corrected typographical errors, in V1·0
Protocol version 3·0	30/03/16	Adds qualitative paramedic substudy
Protocol: submitted for publication	03/05/17	International Journal Stroke: published 01/08/17
Funding request for enhanced recruitment	21/04/17	BHF
Protocol version 4·5	09/08/17	Amends qualitative paramedic substudy. Note No protocol version 4·0.
Statistical analysis plan: submitted for publication	10/10/17	European Stroke Journal: accepted 03/01/18, published 01/02/18. Detailed hierarchical analysis (replacing the plan given in the protocol and published protocol)
Interim analysis by DMC	11/10/17	
Participant 850 recruited	22/11/17	Recruitment and funding extended in view of high mimic rate
Protocol version 5·0	05/02/18	Detailed high mimic rate. Incorrectly gave intended sample size as 1300
Participant 1050 recruited	02/03/18	Recruitment continued to use remaining trial funding to end May 2018
Last patient (1149) recruited	23/05/18	
Baseline patient characteristics: submitted for publication	20/08/18	International Journal Stroke: published 26/11/18
Last day 90 follow-up	12/10/18	
End of trial funding	31/10/18	
Hard data lock	08/01/19	

Table 2. Adherence and reasons for non-adherence, in confirmed stroke/TIA patients (cohort 1/target disease population) and all patients (cohort 2/intention-to-treat). Data are number (%).

	**Confirmed**	**stroke/TIA**	**All**	
	GTN	Sham	GTN	Sham
Participants with data	434	418	568	581
**Adherence (%), received**				
First treatment [a]	432 (>99)	417 (>99)	565 (99)	580 (>99)
One or more patches	432 (>99)	417 (>99)	565 (99)	580 (>99)
At least first 2 days of treatment	287 (66)	284 (68)	311 (55)	320 (55)
All 4 days treatment	185 (43)	197 (47)	198 (35)	210 (36)
No randomised treatment	2 (<1)	1 (<1)	3 (1)	2 (<1)
**Reasons for non-adherence (%)** [b]				
Non-stroke diagnosis initially [c]	30 (7)	29 (7)	123 (22)	133 (23)
Serious adverse event	2 (<1)	2 (<1)	2 (<1)	2 (<1)
Adverse event (not an SAE)	2 (<1)	0 (0)	2 (<1)	0 (0)
Discharged before day 2	22 (5)	16 (4)	25 (4)	21 (4)
Participant/proxy refused patch	5 (1)	0 (0)	6 (1)	2 (<1)
Medical decision to stop treatment	29 (7)	20 (5)	30 (5)	21 (4)
Procedural error	20 (5)	25 (6)	20 (4)	26 (5)
Trial medication missing / not available	11 (3)	9 (2)	11 (2)	9 (2)
Died	8 (2)	8 (2)	8 (1)	9 (2)
Others	15 (3)	21 (5)	18 (3)	23 (4)

[a] Patients receiving at least the first two days of treatment are considered to have been adherent to treatment
[b] Reasons for non-adherence are not mutually exclusive
[c] Participant in cohort 1 initially diagnosed as non-stroke and patch withdrawn before final diagnosis of stroke made

Table 3. Blinding to treatment at day 90 follow-up, all patients (cohort 2/intention-to-treat). Data are number (%).

Treatment	GTN	Sham
Did not know	388 (96%)	407 (97%)
Guessed GTN	12 (3%)	10 (2%)
Guessed sham	4 (1%)	2 (1%)

GTN: glyceryl trinitrate
Excludes those who died or would not answer the question

Table 4. Additional pre-specified outcome analyses at days 4 and 90 by treatment group, confirmed stroke/TIA patients (cohort 1/target disease population). Data are number (%), median [interquartile quartile range] or mean (standard deviation). Comparison by binary logistic regression (BLR), Cox proportional hazards regression (Cox), ordinal logistic regression (OLR), or multiple linear regression (MLR), with adjustment for age, sex, pre-morbid mRS, FAST, pre-treatment systolic BP, index event (haemorrhagic stroke, ischaemic stroke, TIA, mimic), time to randomisation, and reperfusion therapy (alteplase, intra-arterial therapy, none) (unless stated). The effect of treatment for GTN versus sham is shown as adjusted common odds ratio (acOR), adjusted hazard ratio (aHR), or adjusted difference in means (aDIM), with 95% confidence intervals.

Outcome (%)	N	GTN	Sham	acOR/aHR/aDIM (95% CI)	p-value
Day 4 (or discharge)					
Confirmed stroke/TIA					
Infection	843	73 (17)	70 (17)	1·02 (0·70, 1·49)	0·91
Glasgow coma scale (/15)	697	13·2 (3·4)	13·4 (3·3)	-0·15 (-0·59, 0·29)	0·51
NIHSS (/43) [a]	565	10·3 (11·7)	9·3 (11·3)	0·89 (-0·73, 2·51)	0·28
Patients with an SAE	849	110 (25)	90 (22)	1·31 (0·93, 1·85)	0·13
Neurological deterioration, clinical	845	58 (13)	61 (15)	0·90 (0·60, 1·36)	0·62
Symptomatic VTE [b]	852	1 (<1)	0 (0)	-	1·00
Use of labetalol	839	51 (12)	44 (11)	1·06 (0·65, 1·74)	0·80
After ischaemic stroke or TIA					
Symptomatic HTI [c]	702	7 (2)	12 (4)	0·43 (0·15, 1·19)	0·10
Post-thrombolysis HTI	282	5 (3)	11 (8)	0·35 (0·11, 1·10)	0·073
Transient ischaemic attack	702	8 (2)	6 (2)	1·05 (0·30, 3·68)	0·94
Major extra-cranial bleeding	702	1 (<1)	1 (<1)	0·22 (0·00, 11·33)	0·45
Symptomatic recurrence	699	3 (1)	9 (3)	0·27 (0·07, 1·05)	0·059
After intracerebral haemorrhage					
Symptomatic recurrence	141	1 (1)	2 (3)	3·50 (0·01, 1804)	0·69
Day 90					
Quality of life, EQ-VAS (/100) [a]	742	45·4 (33·9)	46·7 (31·9)	-0·93 (-5·10, 3·24)	0·66
Disability, Barthel index <60 [a]	795	171 (42)	162 (42)	1·13 (0·80, 1·58)	0·49
Cognition, tMMSE [a]	446	10·5 (10)	11·1 (9·7)	-0·71 (-2·14, 0·71)	0·32
Cognition, Animal naming [a]	440	8·5 (9·4)	9·4 (9·9)	-1·04 (-2·49, 0·41)	0·16

[a] Death assigned: BI -5, animal naming -1, EQ-VAS -1, home time -1, tMMSE -1, TICS-M -1, EQ-5D HUS 0, GCS 2, NIHSS 43, ZDS 102·5
[b] VTE; venous thromboembolism = deep vein thrombosis and/or pulmonary embolism
[c] Haemorrhagic transformation of infarct (HTI) according to IST-3 definition [27]

Table 5. In hospital management and treatment, confirmed stroke/TIA patients (cohort 1/target disease population). Data are number (%) or median [interquartile quartile range]. Comparison by binary logistic regression (BLR) or multiple linear regression (MLR), with adjustment for age, sex, pre-morbid mRS, FAST, pre-treatment systolic BP, index event (haemorrhagic stroke, ischaemic stroke, TIA, mimic), time to randomisation, and reperfusion therapy (alteplase, intra-arterial therapy, none) (unless stated). The effect of treatment for GTN versus sham is shown as adjusted odds ratio (aOR), or adjusted difference in means (aDIM), with 95% confidence interval.

**Outcome (%)**	**N**	**GTN**	**Sham**	**aOR/aDIM (95% CI)**	**p-value**
Ischaemic stroke or TIA					
Intravenous alteplase	706	150 (42)	135 (39)	10·91 (0·12, 977·7)	0·30
Door-to-needle time (mins)	285	49 [40, 66]	54 [43, 69]	3.5 (-1, 8)	0·14
Thrombectomy	698	7 (2)	17 (5)	0·35 (0·14, 0·87)	0·024
Carotid endarterectomy	698	1 (<1)	2 (1)	0·11 (0·00, 6·48)	0·28
Stroke or TIA					
Hemicraniectomy	839	2 (<1)	2 (<1)	0·83 (0·08, 9·01)	0·88
Other surgery	839	5 (1)	3 (1)	1·75 (0·33, 9·33)	0·51
Neurosurgical Unit	842	10 (2)	13 (3)	0·90 (0·35, 2·27)	0·82
Intensive care unit	842	15 (4)	14 (3)	1·06 (0·46, 2·46)	0·89
Ventilation	841	16 (4)	6 (1)	3.13 (1·12, 8·80)	0·030
Acute Stroke Unit	847	357 (83)	348 (84)	1·00 (0·67, 1·47)	0·98
Stroke Rehabilitation Unit	848	119 (28)	141 (34)	0·74 (0·55, 1·01)	0·055
Physiotherapy	845	327 (76)	336 (81)	0·76 (0·52, 1·10)	0·15
Occupational therapy	843	315 (74)	314 (76)	0·92 (0·65, 1·30)	0·62
Speech therapy	842	258 (60)	275 (66)	0·76 (0·56, 1·05)	0·10

aDIM: adjusted difference in means; aOR: adjusted odds ratio

Table 6. Serious adverse events up to day 90 by treatment group, all patients (cohort 2/intention-to-treat). Data are number (%) of affected patients. Comparison by unadjusted binary logistic regression

Cause	All			Fatal		
	GTN	Sham	p-value	GTN	Sham	p-value
Total Number of patients	568	581		568	581	
Total with at least 1 SAE	188 (33)	170 (29)	0.16	105 (18)	98 (17)	0.47
Range of SAEs per participant	[1-4]	[1-4]		-	-	
**SAE type**						
Cardiovascular	29 (5)	16 (3)	0.043	9 (2)	3 (1)	0.091
Neurological	107 (19)	91 (16)	0.15	58 (10)	51 (9)	0.41
Respiratory	49 (9)	42 (7)	0.38	24 (4)	20 (3)	0.49
Gastrointestinal	3 (1)	3 (1)	0.98	2 (<1)	0 (0)	
Genito-urinary	9 (2)	5 (1)	0.27	1 (<1)	1 (<1)	0.99
Haematological/ immunological	1 (<1)	0 (0)		0 (0)	0 (0)	
Metabolic / endocrine	1 (<1)	0 (0)		1 (<1)	0 (0)	
Musculoskeletal/cutaneous	2 (<1)	5 (1)	0.28	0 (0)	0 (0)	
Infection	60 (11)	54 (9)	0.47	25 (4)	21 (4)	0.50
Miscellaneous	8 (1)	23 (4)	0.011	6 (1)	20 (3)	0.010
**Relationship with IMP**						
Total serious adverse events	230	204		105	98	
Definite	4 (2)	1 (<1)	0.25	0 (0)	0 (0)	0.19
Probable	85 (37)	63 (31)		36 (34)	23 (23)	
Possible	40 (17)	50 (25)		13 (12)	21 (21)	
Improbable	37 (16)	31 (15)		18 (17)	15 (15)	
Not related	64 (28)	59 (29)		38 (36)	39 (40)	

There were no SUSARs. SAEs are not mutually exclusive.

Table 7. Imaging at admission and days 2-4, confirmed stroke/TIA patients (cohort 1/target disease population)· Data are number (%), median [interquartile quartile range] or mean (standard deviation). The effect of treatment for GTN versus sham are shown as difference in means (DIM), with 95% confidence intervals, with comparisons performed using binary logistic regression or multiple linear regression.

**Outcome (%)**	**Admission**			**acOR/aOR/aDIM (95% CI)**	**p**	**Day 2-4**			**acOR/aOR/aDIM (95% CI)**	**p**
	N	GTN N=375	Sham N=374			N	GTN N=263	Sham N=260		
All										
Normal Scan	841	4 (1)	0 (0)			641	1 (<1)	0 (0)		
Ischaemic stroke										
Scan type										
CT	593	298 (100)	294 (>99)			519	223 (86)	226 (87)	0·88 (0·53, 1·46)	0·62
MRI	593	0 (0)	1 (<1)			519	37 (14)	33 (13)	1·14 (0·69, 1·88)	0·62
Infarct size	590			1·15 (0·82, 1·61)	0·41	516			1·07 (0·78, 1·46)	0·68
Very large		0 (0)	1 (<1)				7 (3)	5 (2)		
Large		9 (3)	7 (2)				41 (16)	34 (13)		
Medium		34 (12)	29 (10)				54 (21)	64 (25)		
Small		61 (21)	58 (20)				92 (36)	85 (33)		
None visible		191 (65)	200 (68)				65 (25)	69 (27)		
Infarct swelling	590	68 (23)	56 (19)	1·28 (0·86, 1·90)	0·23	518	144 (56)	149 (58)	0·92 (0·65, 1·31)	0·66
Infarct mass effect	590	0 [0,0]	0 [0,0]	1·3 (0·87, 1·93)	0·20	518	1 [0,2]	1 [0,2]	0·96 (0·7, 1·32)	0·80
Post-alteplase HTI						281	5 (3)	11 (8)	0·38 (0·13, 1·13)	0·082
Intracerebral haemorrhage										
Scan type										
CT	144	73 (100)	71 (100)			97	52 (98)	43 (98)	1·21 (0·07, 19·91)	0·89
MRI	144	0 (0)	0 (0)			97	1 (2)	1 (2)	0·83 (0·05, 13·61)	0·89
ICH size	142	2 [2,3]	2 [1,3]	1·95 (1·07, 3·58)	0·030	97	2 [1,3]	1 [1,2·5]	2·05 (0·97, 4·34)	0·060
<3 cm		17 (24)	29 (41)				19 (36)	23 (52)		
3-5 cm		24 (33)	21 (30)				11 (21)	10 (23)		
5-8 cm		23 (32)	13 (19)				17 (32)	8 (18)		
>8 cm		8 (11)	7 (10)				6 (11)	3 (7)		
Haematoma mass effect	144	2 [2,4]	2 [2,2]	2·42 (1·26, 4·68)	0·0083	97	2 [2,3]	2 [2,2]	1·6 (0·74, 3·49)	0·23

HTI: haemorrhagic transformation of infarct; ICH: intracerebral haemorrhage

Table 8. Protocol violations (pre-defined) by treatment group, all patients (cohort 2/intention-to-treat). Data are number (%)

Violation (%)	GTN	Sham	P value
**In ambulance**			
Onset to randomisation >4 hours	7 (1)	7 (1)	1·00
FAST score or 0 or 1	5 (1)	6 (1)	1·00
Patient from nursing home	4 (1)	2 (<1)	0·45
Systolic blood pressure <120 mmHg	2 (<1)	2 (<1)	1.00
Failure to obtain primary consent	0 (0)	1 (<1)	1·00
Patient did not receive first GTN/sham patch	1 (<1)	0 (0)	0·49
**In hospital**			
Participant did not receive second GTN/sham patch [a]	17 (3)	16 (3)	0·86
Failure to obtain secondary consent, when applicable	4 (1)	5 (1)	1·00
Subsequent randomisation into another trial	0 (0)	1 (<1)	1·00
**Coordinating centre**			
Day 90 follow-up performed day <83 or >104	5 (1)	7 (1)	0·77
Any other violation of the trial protocol	14 (2)	17 (3)	0·72

10 participants had more than one protocol violation
[a] As reported by Investigators – some non-adherence was not reported

Figure 1. Consort flow diagram of patient randomisation, outcome, and losses to follow-up for confirmed stroke/TIA patients (cohort 1/target disease population). Data are number (%).

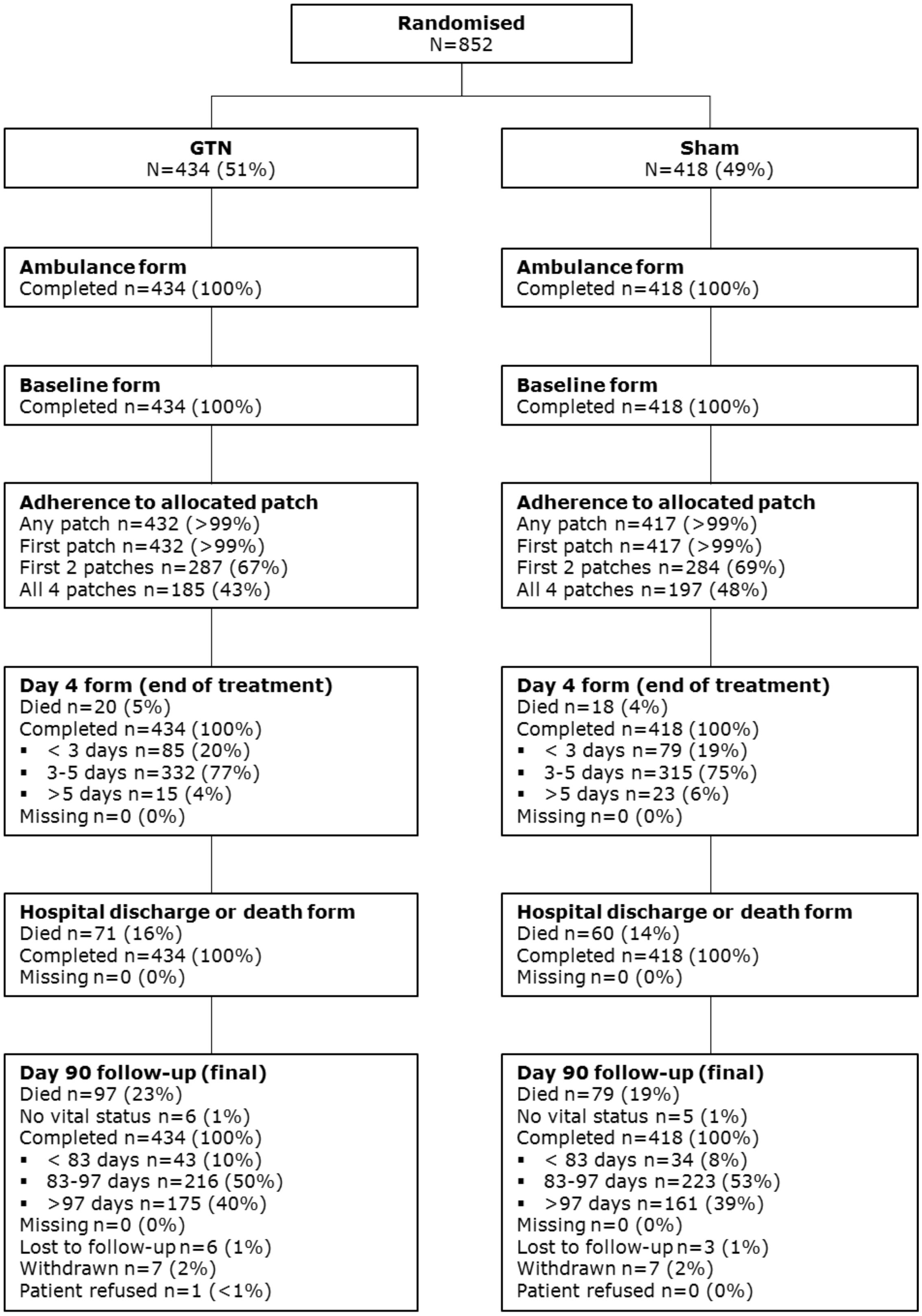

Figure 2. Course of systolic and diastolic blood pressure over 4 days of treatment, confirmed stroke/TIA patients (cohort 1/target disease population). Data are mean (standard deviation) [number]. Comparison by ANOVA with adjustment for baseline blood pressure.

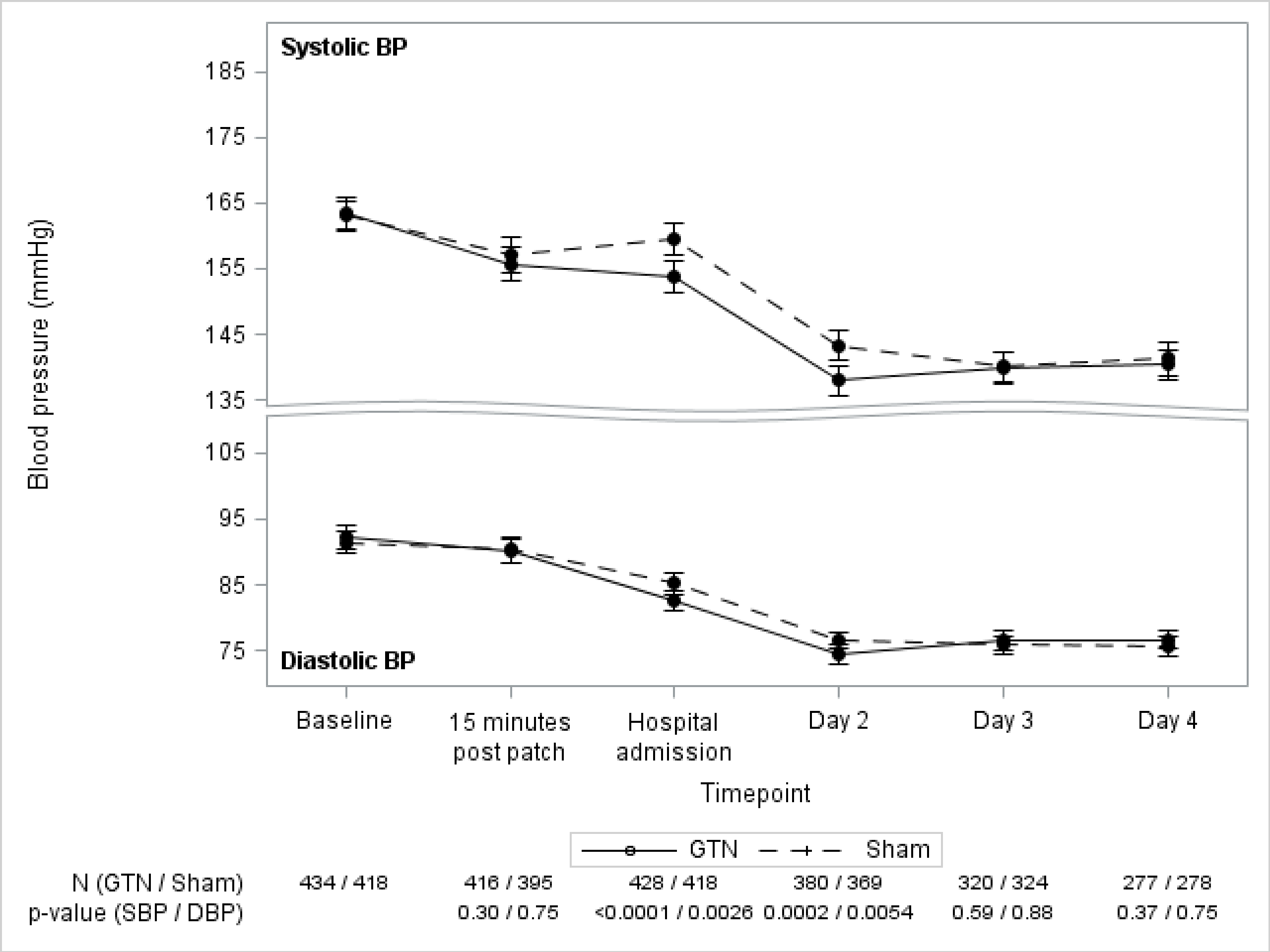

Figure 3. Course of systolic and diastolic blood pressure over 4 days of treatment, all patients (cohort 2/intention-to-treat). Data are mean (standard deviation) [number]. Comparison by ANCOVA with adjustment for baseline blood pressure.

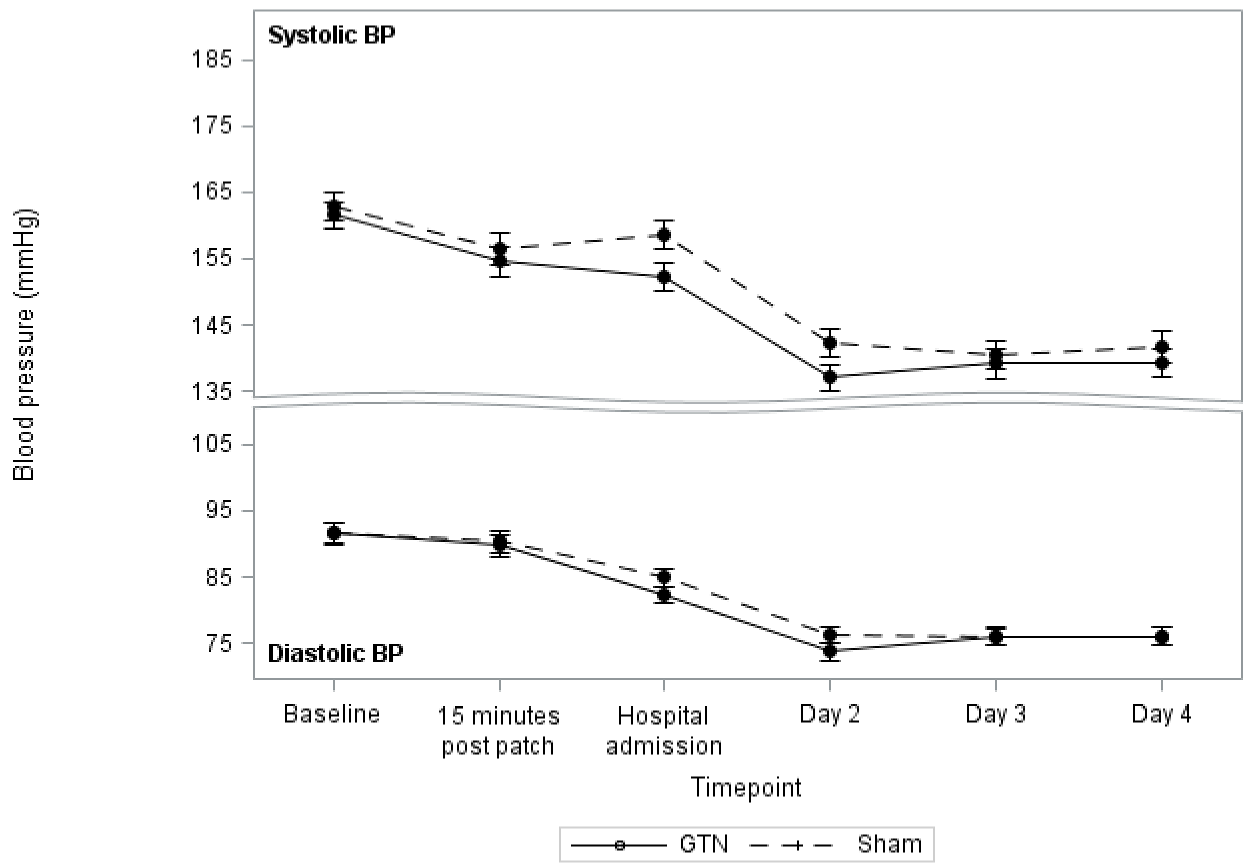

	Baseline	15 minutes post patch	Hospital admission	Day 2	Day 3	Day 4
N (GTN / Sham)	568 / 579	541 / 548	551 / 567	453 / 461	372 / 390	318 / 330
p-value (SBP / DBP)		0.55 / 0.73	<0.0001 / 0.0012	0.0002 / 0.0006	0.45 / 0.80	0.12 / 0.78

Figure 4. Forest plot of effect of treatment on modified Rankin Scale at day 90 in *post hoc* subgroups defined after treatment and admission to hospital, confirmed stroke/TIA patients (cohort 1/target disease population). Data are odds ratio (95% confidence intervals) and interaction test. Comparison by ordinal logistic regression adjusted for age, sex, pre-morbid mRS, FAST, pre-treatment systolic BP, index event (haemorrhagic stroke, ischaemic stroke, TIA, mimic), and time to treatment.

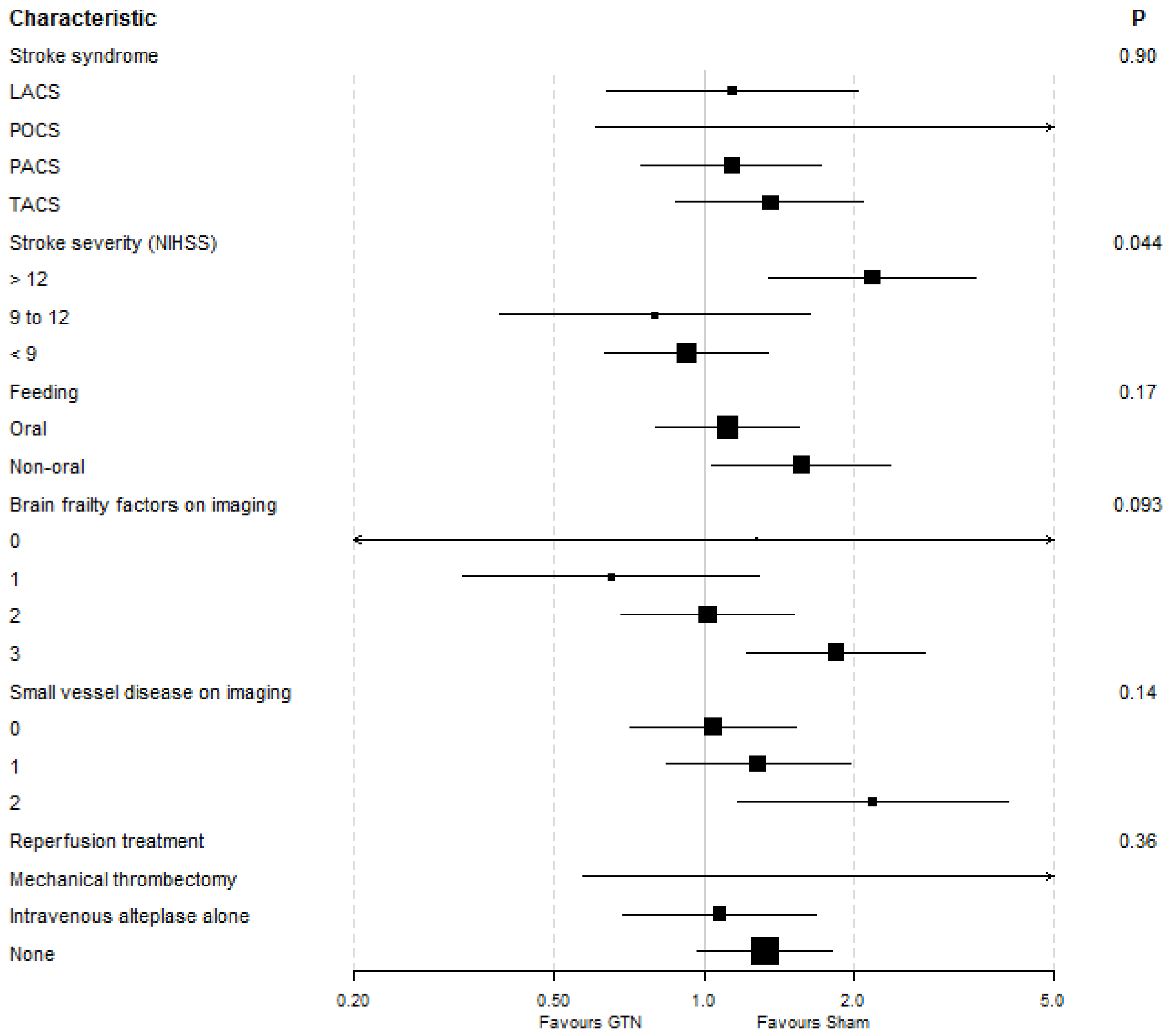

Figure 5. Distribution of modified Rankin Scale (mRS) at day 90 for glyceryl trinitrate (GTN) versus sham, all patients (cohort 2/intention-to-treat). Comparison by ordinal logistic regression adjusted for age, sex, pre-morbid mRS, FAST score, pre-treatment systolic BP, index event (intracerebral haemorrhage, ischaemic stroke, transient ischaemic attack, mimic) and time to treatment.

Adjusted common odds ratio 1·04 (95% confidence intervals 0·84–1·29), p=0·69

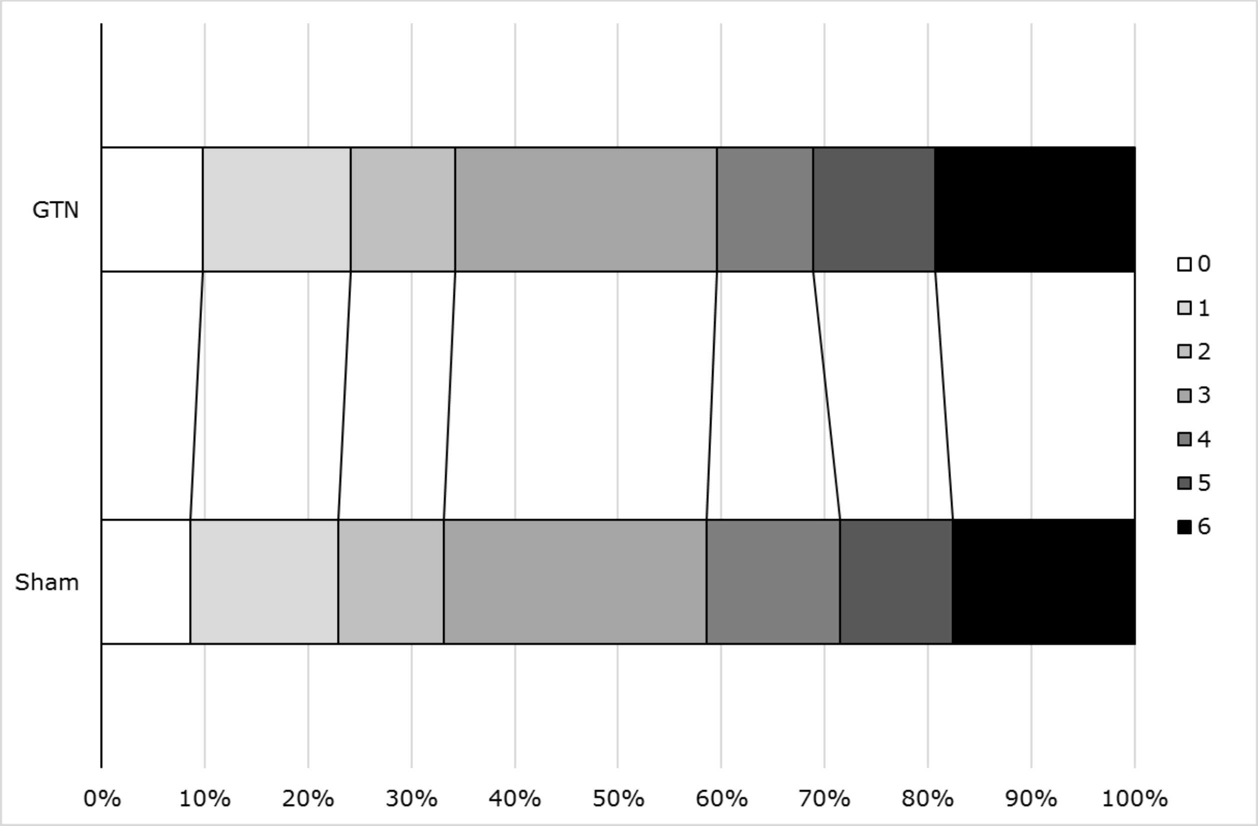

Figure 6. Forest plot of effect of treatment on modified Rankin Scale at day 90 in pre-specified subgroups defined before treatment or on admission to hospital, all patients (cohort 2/intention-to-treat). Data are odds ratio (95% confidence intervals) and interaction test. Comparison by ordinal logistic regression adjusted for age, sex, pre-morbid mRS, FAST, pre-treatment systolic BP, index event (haemorrhagic stroke, ischaemic stroke, TIA, mimic), and time to randomisation.

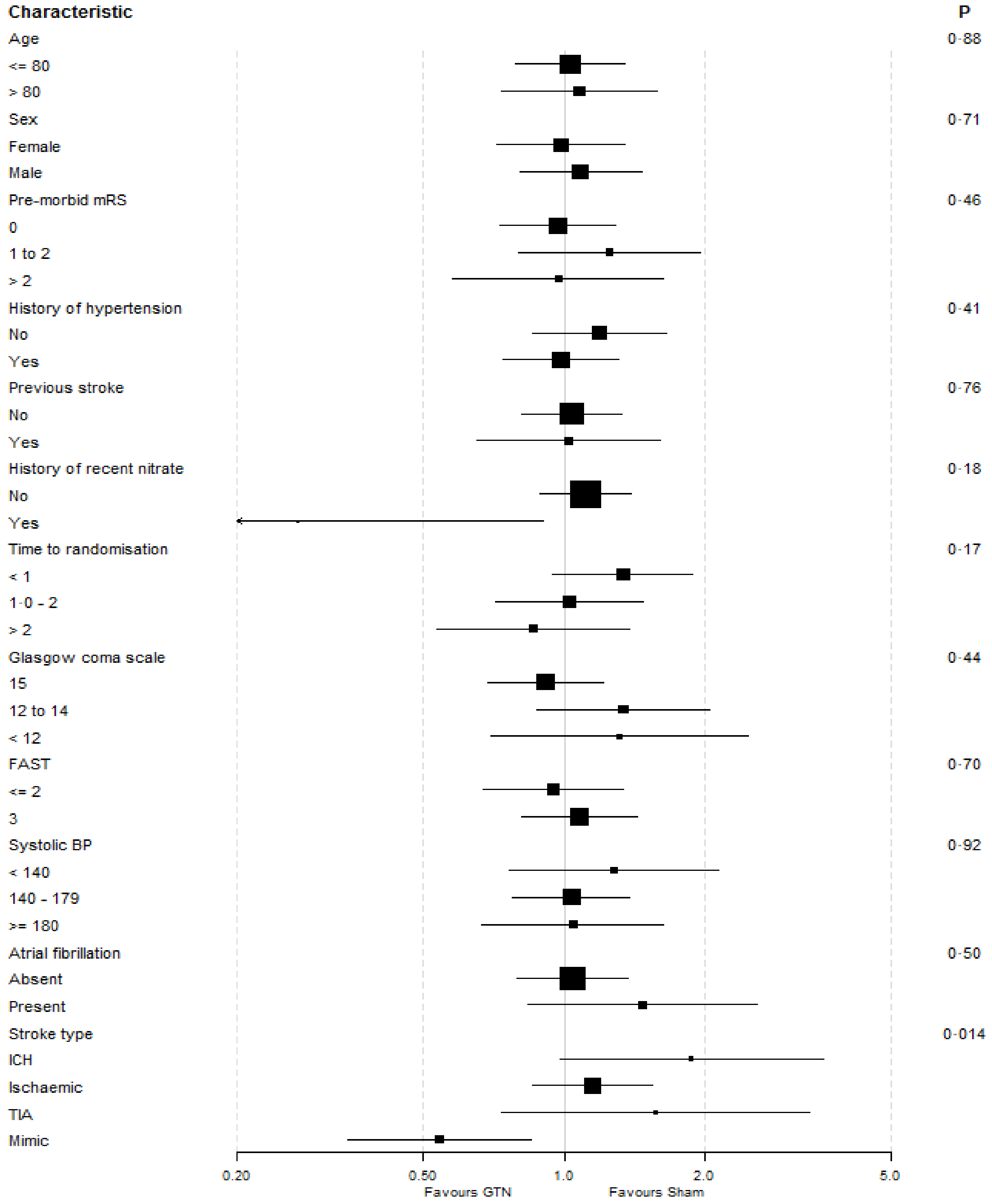

Figure 7. Forest plot of outcomes included in the global analysis (modified Rankin scale, Barthel index, Euro-Qol-5D, Zung depression scale, telephone interview cognition scale-modified, analysed by Wei-Lachin test), confirmed stroke/TIA patients (cohort 1/target disease population).

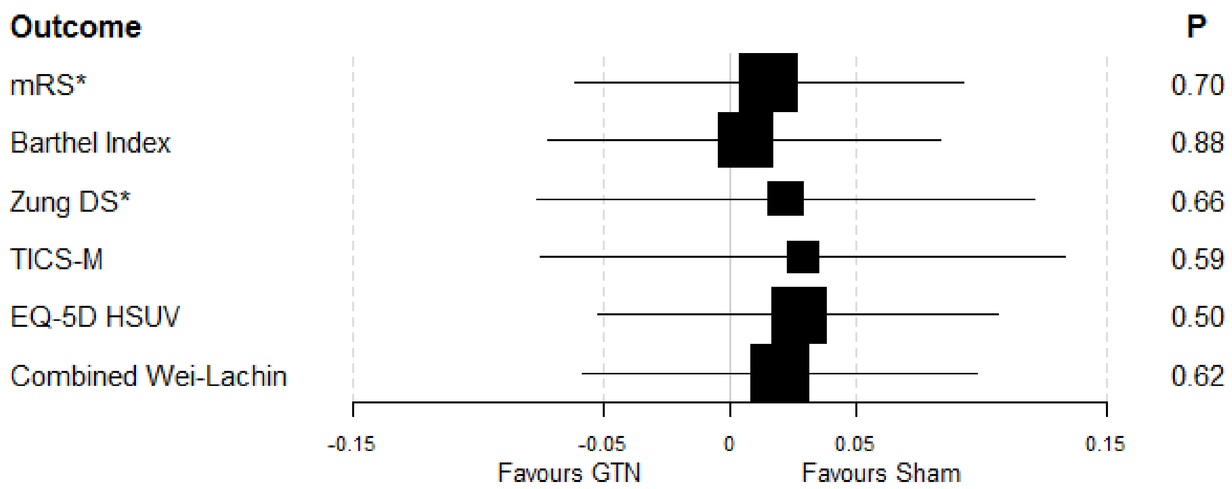

*The scores for the modified Rankin scale and Zung depression score are inverted so that they have the same direction of severity as the Barthel index, telephone interview cognition scale-modified, and Euro-quality of life 5-dimension 3-level/health status utility value

Figure 8. Cumulative case fatality during the 90 days of follow-up after randomisation, confirmed stroke/TIA patients (cohort 1/target disease population). Comparison of GTN versus sham by Cox regression with adjustment for age, sex, pre-morbid mRS, FAST, pre-treatment systolic BP, index event (haemorrhagic stroke, ischaemic stroke, TIA, mimic), time to randomisation and reperfusion therapy (alteplase, intra-arterial therapy, none).

Adjusted hazard ratio 1·24 (95% confidence intervals 0·91–1·68), p=0·17

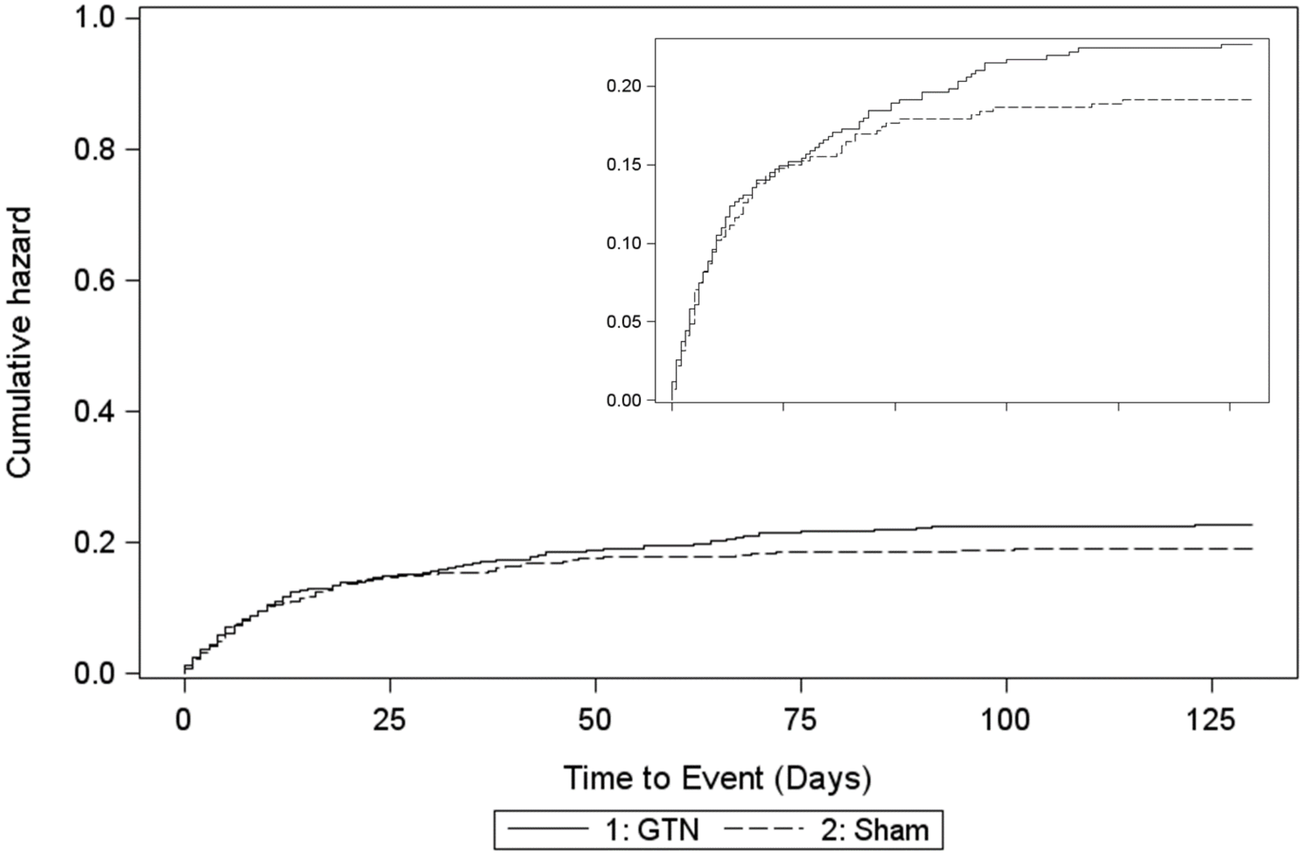

1: GTN	428	364	347	336	332	331
2: Sham	413	352	340	336	335	334

Figure 9. Cumulative case fatality during the 90 days of follow-up after randomisation, all patients (cohort 2/intention-to-treat). Comparison of GTN versus sham by Cox regression with adjustment for age, sex, pre-morbid mRS, FAST, pre-treatment systolic BP, index event (haemorrhagic stroke, ischaemic stroke, TIA, mimic), time to treatment and reperfusion therapy (alteplase, intra-arterial therapy, none).

Adjusted hazard ratio 1·11 (95% confidence intervals 0·84–1·47), p=0·47

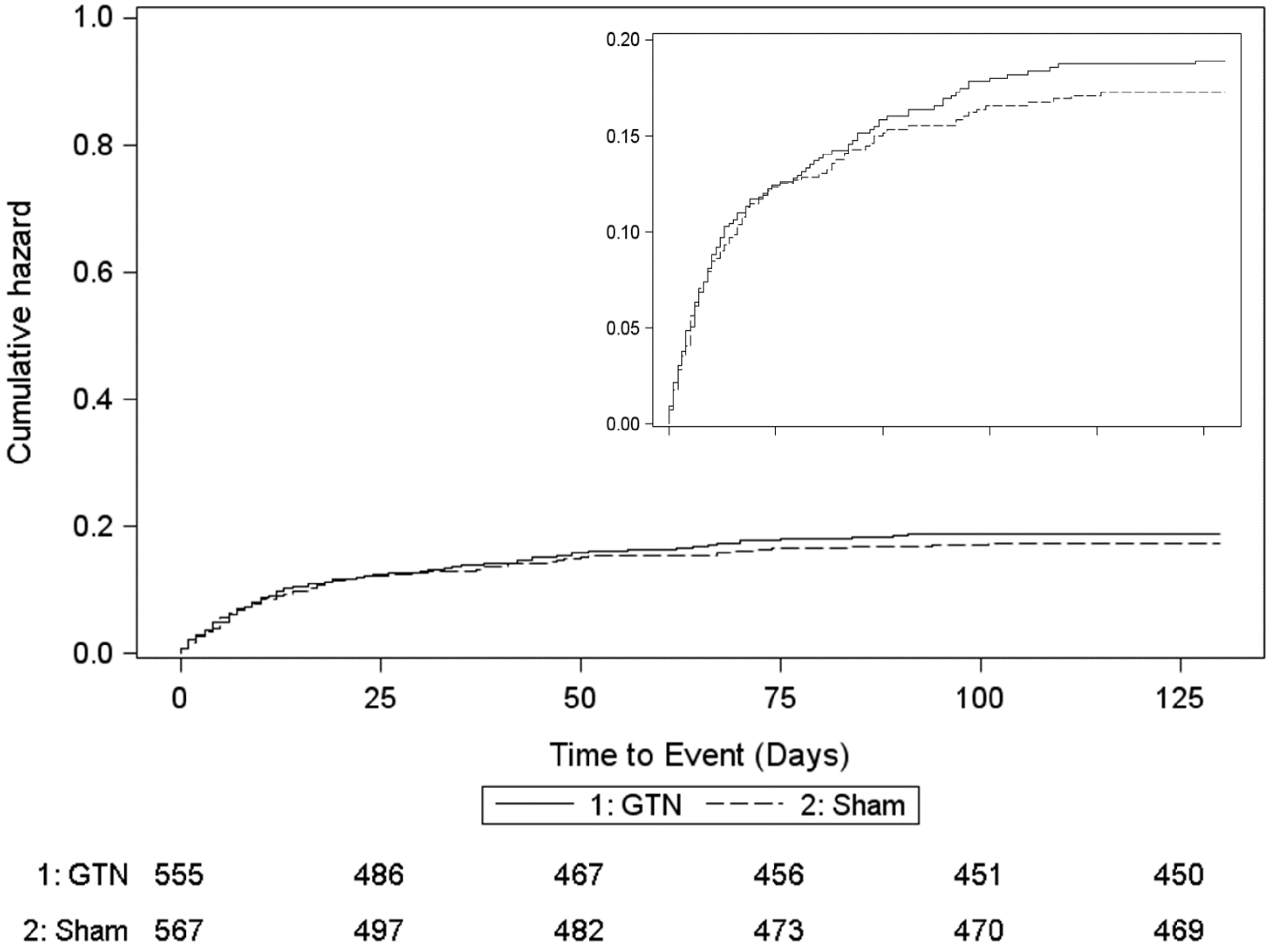

Figure 10. Meta-analysis of effect of treatment in patients treated within 6 hours of randomisation. Analysis using unadjusted data. RIGHT-2 patients are those with confirmed stroke/TIA (cohort 1/target disease population) so as to match those in RIGHT and ENOS-early. Data are numbers and odds ratio (95% confidence interval).

A. Death or dependency (mRS >2)

	GTN		Control			Odds Ratio	
**Study or Subgroup**	**Events**	**Total**	**Events**	**Total**	**Weight**	**M-H, Random, 95% CI**	**Year**
**1.1.1 Overall**							
RIGHT 2013	12	24	11	15	4.9%	0.36 [0.09, 1.47]	2013
ENOS-early 2015	74	144	78	129	34.1%	0.69 [0.43, 1.12]	2015
RIGHT-2 2018	257	364	258	358	61.0%	0.93 [0.67, 1.29]	2018
**Subtotal (95% CI)**		**532**		**502**	**100.0%**	**0.80 [0.59, 1.10]**	
Total events	343		347				
Heterogeneity: Tau² = 0.01; Chi² = 2.38, df = 2 (P = 0.30); I² = 16%							
Test for overall effect: Z = 1.37 (P = 0.17)							
**Total (95% CI)**		**532**		**502**	**100.0%**	**0.80 [0.59, 1.10]**	
Total events	343		347				
Heterogeneity: Tau² = 0.01; Chi² = 2.38, df = 2 (P = 0.30); I² = 16%							
Test for overall effect: Z = 1.37 (P = 0.17)							
Test for subgroup differences: Not applicable							

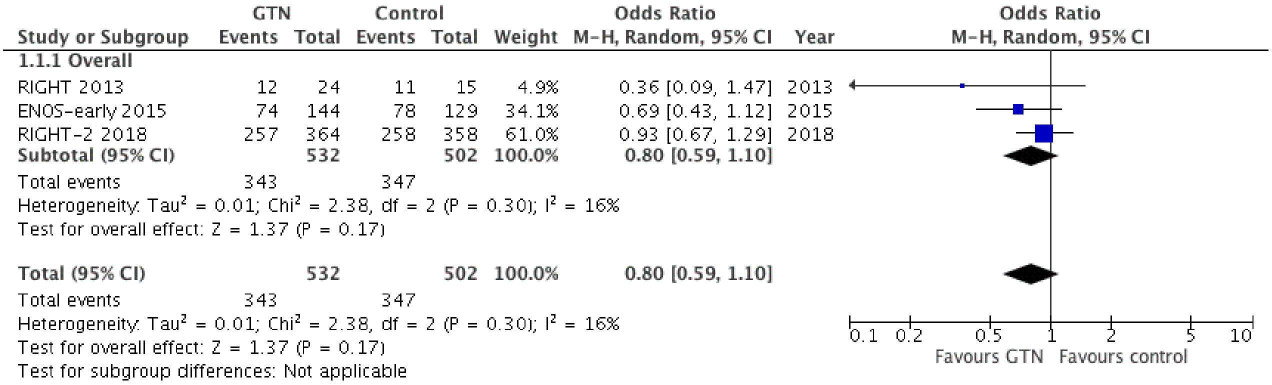

B. Death

	NO donor		Control			Odds Ratio
**Study or Subgroup**	**Events**	**Total**	**Events**	**Total**	**Weight**	**M-H, Random, 95% CI**
**1.9.1 Overall**						
ENOS-early 2015	11	144	26	129	35.8%	0.33 [0.15, 0.69]
RIGHT 2013	3	24	6	15	24.1%	0.21 [0.04, 1.05]
RIGHT-2 2018	95	369	75	359	40.2%	1.31 [0.93, 1.85]
**Subtotal (95% CI)**		**537**		**503**	**100.0%**	**0.52 [0.16, 1.72]**
Total events	109		107			
Heterogeneity: Tau² = 0.91; Chi² = 14.47, df = 2 (P = 0.0007); I² = 86%						
Test for overall effect: Z = 1.08 (P = 0.28)						
**Total (95% CI)**		**537**		**503**	**100.0%**	**0.52 [0.16, 1.72]**
Total events	109		107			
Heterogeneity: Tau² = 0.91; Chi² = 14.47, df = 2 (P = 0.0007); I² = 86%						
Test for overall effect: Z = 1.08 (P = 0.28)						
Test for subgroup differences: Not applicable						

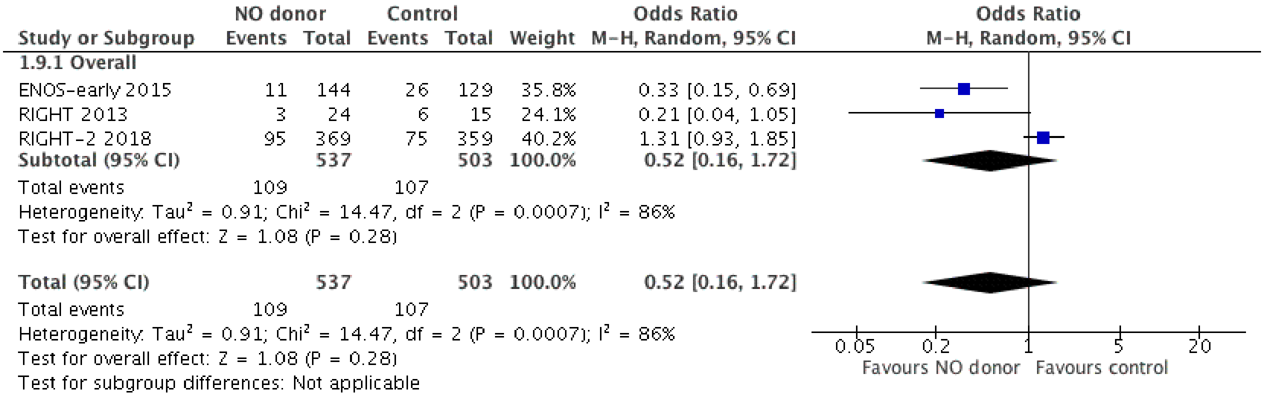

Supplement: Supplementary appendix [file mmc1.pdf]
